# Mercury Clathration-Driven Phase Transition in a Luminescent Bipyrazolate Metal–Organic Framework: A Multitechnique Investigation

**DOI:** 10.1021/acs.chemmater.2c03801

**Published:** 2023-03-22

**Authors:** Marco Moroni, Luca Nardo, Angelo Maspero, Guglielmo Vesco, Marco Lamperti, Luca Scapinello, Rebecca Vismara, Jorge A. R. Navarro, Damiano Monticelli, Andrea Penoni, Massimo Mella, Simona Galli

**Affiliations:** † Dipartimento di Scienza e Alta Tecnologia, Università degli Studi dell’Insubria, Via Valleggio 11, 22100 Como, Italy; ‡ Departamento de Química Inorgánica, Universidad de Granada, Av. Fuentenueva S/N, 18071 Granada, Spain; § Consorzio Interuniversitario Nazionale per la Scienza e Tecnologia dei Materiali, Via Giusti 9, 50121 Firenze, Italy

## Abstract

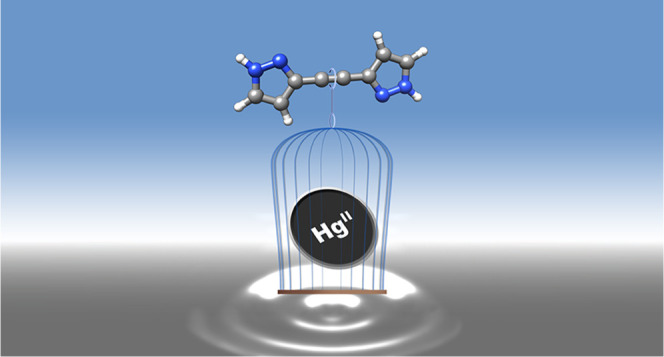

Mercury is one of the most toxic heavy metals. By virtue of its triple bond, the novel ligand 1,2-bis(1*H*-pyrazol-4-yl)ethyne (H_2_BPE) was expressly designed and synthesized to devise metal–organic frameworks (MOFs) exhibiting high chemical affinity for mercury. Two MOFs, Zn(BPE) and Zn(BPE)·*n*DMF [interpenetrated i-Zn and noninterpenetrated ni-Zn·S, respectively; DMF = dimethylformamide], were isolated as microcrystalline powders. While i-Zn is stable in water for at least 15 days, its suspension in HgCl_2_ aqueous solutions prompts its conversion into HgCl_2_@ni-Zn. A multitechnique approach allowed us to shed light onto the observed HgCl_2_-triggered i-Zn-to-HgCl_2_@ni-Zn transformation at the molecular level. Density functional theory calculations on model systems suggested that HgCl_2_ interacts via the mercury atom with the carbon–carbon triple bond exclusively in ni-Zn. Powder X-ray diffraction enabled us to quantify the extent of the i-Zn-to-HgCl_2_@ni-Zn transition in 100–5000 ppm HgCl_2 (aq)_ solutions, while X-ray fluorescence and inductively coupled plasma-mass spectrometry allowed us to demonstrate that HgCl_2_ is quantitatively sequestered from the aqueous phase. Irradiating at 365 nm, an intense fluorescence is observed at 470 nm for ni-Zn·S, which is partially quenched for i-Zn. This spectral benchmark was exploited to monitor in real time the i-Zn-to-HgCl_2_@ni-Zn conversion kinetics at different HgCl_2 (aq)_ concentrations. A sizeable fluorescence increase was observed, within a 1 h time lapse, even at a concentration of 5 ppb. Overall, this comprehensive investigation unraveled an intriguing molecular mechanism, featuring the disaggregation of a water-stable MOF in the presence of HgCl_2_ and the self-assembly of a different crystalline phase around the pollutant, which is sequestered and simultaneously quantified by means of a luminescence change. Such a case study might open the way to new-conception strategies to achieve real-time sensing of mercury-containing pollutants in wastewaters and, eventually, pursue their straightforward and cost-effective purification.

## 1 Introduction

Mercury is one of the most toxic heavy metals.^1^ Largely present in surface and ground waters, through the food chain it can reach the human body and be bioaccumulated, leading to serious diseases involving the nervous, digestive, and immune systems, as well as lungs, kidneys, skin, and eyes.^2,3^ Organomercury compounds, in particular, are the most toxic form in which mercury is present in the environment.^4^ Based on this, the U.S. Environmental Protection Agency has established the maximum contaminant level of mercury in drinking water at 2 ppb.^5^

Several approaches have been developed to sequestrate and/or sense mercury from wastewaters. The most exploited techniques for removal are precipitation,^6^ liquid extraction,^7^ ion exchange,^8^ and adsorption,^9^ which is possibly the most economic and operationally simple. On the other hand, chemical sensing based on luminescence is the most straightforward, hence diffuse, method. Despite the existing approaches, the development of cutting-edge strategies to remove or detect mercury in wastewaters is still a challenge.^10^

The tailorable nature of metal–organic frameworks^11,12^ (MOFs) has prompted investigations on their use to detect or sequestrate heavy metals^13−15^ and, more specifically, mercury.^16,17^ These studies typically focus on maximizing the performance of novel or renowned MOFs at the bulk level, with no or limited attention to the molecular mechanisms underneath the performance itself, which are nonetheless fundamental to rationalize the behavior of the pollutant/MOF system and evidence new-conception strategies, eventually allowing optimized pollutant detection or sequestration.

**Scheme 1:**
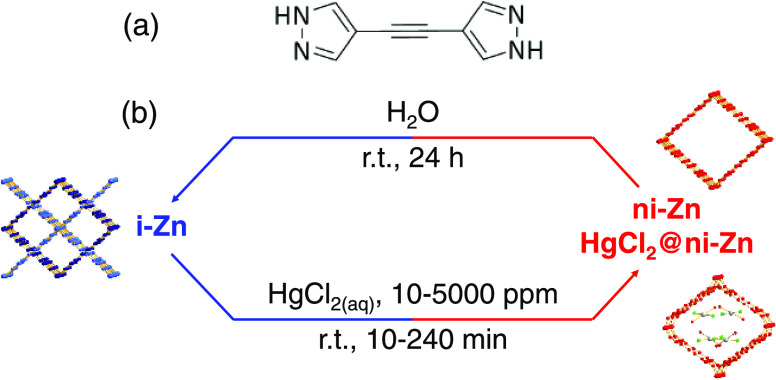
(a) Molecular Structure of the 1,2-Bis(1*H*-pyrazol-4-yl)ethyne Ligand (H_2_BPE). (b) Schematic Representation of HgCl_2_-Triggered Interconversion of Interpenetrated i-Zn into Noninterpenetrated HgCl_2_@ni-Zn

In this context, we report hereafter on the impact of mercury uptake on the transition between the two novel metal–organic frameworks Zn(BPE) and Zn(BPE)·*n*DMF [interpenetrated i-Zn and noninterpenetrated ni-Zn·S, respectively; H_2_BPE = 1,2-bis(1*H*-pyrazol-4-yl)ethyne; DMF = dimethylformamide]. Adopting a multitechnique approach which combines in silico structure modeling with laboratory powder X-ray diffraction (PXRD), N_2_ adsorption, X-ray fluorescence (XRF), inductively coupled plasma-mass spectrometry (ICP-MS) and electronic-state transition spectroscopy (UV–vis absorption and fluorescence), we performed a comprehensive study of the HgCl_2_/Zn(BPE) system, shedding light, at the molecular level, on a peculiar phase transition prompted by HgCl_2_ clathration to yield HgCl_2_@ni-Zn (Scheme 1). To the best of our knowledge, such a peculiar conversion between two MOFs, featuring dissolution of the pristine phase and self-assembly of the other one around a specific guest, has never been reported before.

## 2 Experimental Section

### 2.1 Materials and Methods

Unless otherwise stated, all solvents and reagents were used as obtained from commercial suppliers, without further purification. All reactions involving air- and/or moisture-sensitive materials were carried out under inert atmosphere, using the standard Schlenk-line technique. IR spectra were acquired in the attenuated total reflectance mode or in nujol in Fourier transform mode over the range 4000–600 cm^–1^ with a Nicolet iS10 instrument. In the following, the IR band intensities are denoted as: vs = very strong, s = strong, m = medium, w = weak, and b = broad. Solution ^1^H and ^13^C(APT) NMR spectra were recorded at 400 and 100 MHz, respectively, on a Bruker Avance 400 spectrometer. NMR data are reported as follows: chemical shifts (in ppm and referenced to internal tetramethylsilane), integration, multiplicity (s = singlet, d = doublet, t = triplet, q = quartet, m = multiplet) and coupling constant (in Hz). Elemental composition analyses (C, H, N %) were performed with a PerkinElmer CHN Analyser 2400 Series II instrument. Thermogravimetric analysis and differential scanning calorimetry were performed simultaneously under N_2_ atmosphere over the temperature range 303–873 K, with a heating rate of 10 K min^–1^, employing a Netzsch STA 409 PC Luxx instrument. Gas chromatography–mass spectrometry analyses were performed on a ThermoquestTraceGC instrument equipped with a 30 m DB5 silica column coupled with a FinniganTraceMS quadrupolar mass analyzer. X-ray fluorescence analyses were carried out in air and at room temperature on powdered batches (∼10 mg for each sample) or mother liquors (∼2 mL for each sample) with a Panalytical MINIPAL 2 instrument equipped with a Cr X-ray source. Mercury determination in the mother liquors was performed by a quadrupole iCap Q inductively coupled plasma-mass spectrometer by Thermo Fisher. All of the samples used for the functional studies were characterized by elemental composition analysis, IR spectroscopy and powder X-ray diffraction (see below).

### 2.2 Synthesis of the H_2_BPE Ligand

Details on the synthesis and characterization of the 1,2-bis(1*H*-pyrazol-4-yl)ethyne ligand (H_2_BPE) are reported in Section S1.

### 2.3 Synthesis of Zn(BPE)·DMF, ni-Zn·S

The H_2_BPE ligand (49.8 mg, 0.31 mmol) was dissolved under magnetic stirring in dimethylformamide (DMF) (3.5 mL), previously heated at 383 K. Zn(ClO_4_)_2_·6H_2_O (103.9 mg, 0.28 mmol) was then added under magnetic stirring at 383 K, followed by the dropwise addition of triethylamine (TEA) (1 mL). The reaction mixture was left under magnetic stirring at 383 K for 0.5 h, then at 403 K for 3.5 h. The off-white precipitate thus formed was filtered, washed with DMF and methanol (twice, the first round with 1 and 1 mL, the second round with 1 and 10 mL, respectively), and dried under vacuum at 393 K for 4 h. Yield: 49% (based on Zn^II^). Elemental analysis (%) for C_11_H_11_N_5_OZn (FW = 294.6 a.m.u.), calc.: C = 44.84, H = 3.76, N = 23.77; found: C = 44.75, H = 3.17, N = 23.62. IR(ATR) (ν, cm^–1^) (Figure S1): 1685 (w); 1586 (w); 1382 (vs); 1244 (m); 1159 (w); 1083 (s); 1034 (vs); 851 (s); 638 (s).

### 2.4 Synthesis of i-Zn(BPE), i-Zn

The H_2_BPE ligand (50.5 mg, 0.32 mmol) was dissolved under magnetic stirring in DMF (3.5 mL). Zn(ClO_4_)_2_·6H_2_O (102.3 mg, 0.27 mmol) was then added under magnetic stirring, followed by the dropwise addition of TEA (0.5 mL). The reaction mixture was kept under magnetic stirring at room temperature for 4 h. The off-white precipitate thus formed was filtered, washed with DMF and methanol (twice, the first round with 1 and 1 mL, the second round with 1 and 10 mL, respectively), and dried under vacuum at 393 K for 4 h. Yield: 53% (based on Zn^II^). Elemental analysis (%) for C_8_H_4_N_4_Zn (FW = 221.6 a.m.u.), calc.: C = 43.37, H = 1.82, N = 25.29; found, C = 43.86, H = 2.25, N = 25.01. IR(ATR) (ν, cm^–1^) (Figure S1): 1683 (w); 1585 (w); 1382 (vs); 1245 (m); 1159 (w); 1082 (s); 1033 (vs); 851 (s); 637 (s).

### 2.5 Crystal Structure Determination

Powdered samples (∼50 mg) of ni-Zn·S and i-Zn were placed in the cavity of a 0.2 mm deep silicon free-background sample holder (Assing Srl, Monterotondo, Italy). PXRD data acquisitions were carried out at room temperature on a Bruker AXS D8 Advance vertical-scan θ:θ diffractometer, equipped with a sealed X-ray tube (Cu Kα, λ = 1.5418 Å), a Bruker Lynxeye linear position-sensitive detector, a filter of nickel in the diffracted beam and the following optical components: primary- and diffracted-beam Soller slits (aperture 2.5°), fixed divergence slit (aperture 0.5°), antiscatter slit (aperture 8 mm). The generator was operated at 40 kV and 40 mA. Preliminary PXRD measurements to check the purity and crystallinity of the batches were performed in the 2θ range 3.0–35.0°, with steps of 0.02° and time per step of 1 s. Data acquisitions for the structure characterization were performed in the 2θ range 5.0–105.0°, with steps of 0.02° and an overall scan time of about 12 h. Comparison of the PXRD pattern of ni-Zn·S with that of Zn(BPZ)^18^ [H_2_BPZ = 4,4′-bis(1*H*-pyrazole)] suggested that the two compounds are isoreticular. Nonetheless, the unit cell parameters of the new MOFs were independently retrieved upon indexing: a standard peak search allowed the estimation of the maximum positions of the first 20 low- to medium-angle peaks which, through the Singular Value Decomposition algorithm^19^ available in TOPAS-R v.3,^20^ provided approximate unit cell parameters. The space groups were attributed based on the observed systematic absences. To describe the crystallographically independent portion of the BPE^2–^ ligand and the DMF molecule, rigid bodies were built up using the z-matrix formalism, assigning idealized values to bond distances and angles.^21^ For ni-Zn·S, the metal ion and the ligand, which was initially assumed to be planar, were located according to the crystal structure of Zn(BPZ),^18^ while the position and orientation of the DMF molecule were individuated using the Simulated Annealing approach^22^ available in TOPAS-R v.3. As for i-Zn, the metal ion and the center of mass of the spacer, preliminarily assumed to be planar, were located on proper symmetry elements, while the orientation of the linker was assessed through the Simulated Annealing approach. After the structure determination, instrumental and structural parameters of both ni-Zn·S and i-Zn were collectively refined through the so-called Rietveld method^23^ with TOPAS-R v.3. During the final Rietveld refinement stages, ligand and DMF bond distances (except the C–H and the C=O ones) were refined in restrained ranges of values^24^ defined through a search in the Cambridge Structural Database (v. 2021^25^) for room-temperature crystal structures containing the M(pyrazolate) (M = 3d metal ion) moiety or the DMF molecule. In both MOFs, the ligand was eventually allowed to deviate from planarity: in no case a sensible deviation, concomitant to a significant lowering of the figures of merit, was observed so that the spacer was eventually kept planar. The background was modeled through a Chebyshev-type polynomial function. An isotropic thermal factor (*B*_iso_) was refined for the metal center; the isotropic thermal factor of the other elements was calculated as *B*_iso_(L) = *B*_iso_ + 2.0 Å^2^. The instrumental contribution to the peak profile was modeled by means of the Fundamental Parameters Approach.^26^ The sample contribution to the anisotropic peak broadening was accounted for using second- or fourth-order spherical harmonics. The final Rietveld refinement plots are shown in Figure S2a,b.

#### 2.5.1 Crystal Data for ni-Zn·S, Zn(BPE)·1.2DMF

C_11.6_H_12.4_N_5.2_O_1.2_Zn, FW = 309.2 a.m.u., tetragonal, *P*4_2_/*mmc*, *a* = 11.4879(5) Å, *c* = 7.2691(6) Å, *V* = 959.3(1) Å^3^, *Z* = 16, *Z*′ = 2, ρ = 1.070 g/cm^3^, *F*(000) = 316, *R*_Bragg_ = 0.019, *R*_p_ = 0.068 and *R*_wp_ = 0.087, for 4926 data and 48 parameters in the 6.5–105.0° (2θ) range. CCDC No. 2220416.

#### 2.5.2 Crystal Data for i-Zn, Zn(BPE)

C_8_H_4_N_4_Zn, FW = 221.6 a.m.u., orthorhombic, *Pccm*, *a* = 7.6448(3) Å, *b* = 8.5995(3) Å, *c* = 7.3015(2) Å, *V* = 480.01(3) Å^3^, *Z* = 8, *Z*′ = 2, ρ = 1.533 g/cm^3^, *F*(000) = 220, *R*_Bragg_ = 0.030, *R*_p_ = 0.066 and *R*_wp_ = 0.092, for 4801 data and 40 parameters in the 9.0–105.0° (2θ) range. CCDC No. 2220418.

### 2.6 Variable-Temperature Powder X-ray Diffraction

The thermal behavior of ni-Zn·S and i-Zn was investigated *in situ* by variable-temperature powder X-ray diffraction using a custom-made sample heater (Officina Elettrotecnica di Tenno, Ponte Arche, Italy). A powdered sample (∼20 mg) of the two materials was deposited in an aluminum sample holder and was heated in air with steps of 20 K, acquiring a PXRD pattern at each step in isothermal conditions, adopting the experimental conditions collected in Table 1. Treatment of the data acquired before an appreciable loss of crystallinity was observed was performed by means of a parametric whole powder pattern refinement with the Le Bail method,^27^ available in TOPAS-R v.3.

**Table 1:** Experimental Conditions of the Variable-Temperature PXRD Experiments Carried Out on ni-Zn·S and i-Zn

compound	*T* range (K)	2θ range (deg)
ni-Zn·S	303–723	6.0–28.5
i-Zn	303–763	7.0–27.5

### 2.7 Conversion of Interpenetrated i-Zn, Orthorhombic Polymorph, into Interpenetrated t-ni-Zn, Tetragonal Polymorph

A powdered sample (∼50 mg) of i-Zn was heated in an oven for 30 min at 573 K, the temperature at which the *in situ* variable-temperature powder X-ray diffraction experiment on i-Zn (see Section S4) showed the presence of a new phase. After cooling down to room temperature, a PXRD measurement confirmed the formation of the tetragonal polymorph t-i-Zn. For the sake of completeness, the crystal structure of t-i-Zn was characterized starting from that of the orthorhombic modification, adopting the same methodology described in Section 2.5. The final Rietveld refinement plot is shown in Figure S2c.

#### 2.7.1 Crystal Data for t-i-Zn, Zn(BPE)

C_8_H_4_N_4_Zn, FW = 221.6 a.m.u., tetragonal, *P*4_2_/*mcm*, *a* = 8.0875(7) Å, *c* = 7.2082(6) Å, *V* = 471.47(9) Å^3^, *Z* = 16, *Z*′ = 2, ρ = 1.560 g/cm^3^, *F*(000) = 220, *R*_Bragg_ = 0.007, *R*_p_ = 0.023 and *R*_wp_ = 0.030, for 4771 data and 37 parameters in the 9.6–105.0° (2θ) range. CCDC No. 2220417.

### 2.8 Textural Properties

The N_2_ adsorption isotherms of ni-Zn and i-Zn were measured at 77 K under continuous adsorption conditions using a Micromeritics 3Flex adsorption analyzer, taking advantage of a liquid N_2_ bath with 99.999% purity. Prior to the measurement, powdered samples (ca. 100 mg) of the two MOFs were activated at 393 K under high vacuum (10^–6^ Torr) for 12 h.

### 2.9 Water Stability

In a 50 mL beaker, powdered samples (∼50 mg) of ni-Zn·S or i-Zn were suspended in distilled water (3 mL) at room temperature and under magnetic stirring. At different time lapses, an aliquot of the suspension was dried in air. A PXRD pattern was then acquired with the diffractometer described above in the 2θ range 3.0–35.0°, with a step of 0.02° and a time per step of 1 s, using a 0.2 mm deep aluminum sample holder.

### 2.10 HgCl_2_ Clathration

**Caution**: mercury(II) chloride is highly toxic. i-Zn was suspended in HgCl_2_ aqueous solutions prepared dissolving the solute in distilled water. Different experiments were carried out varying (i) the HgCl_2_ concentration (10, 50, 75, 100, 200, 300, 400, 500, 1000, 2000, 5000 ppm; for the concentration expressed as molarity and the i-Zn/HgCl_2_ molar ratio see Table S1) at a fixed suspension time (60 min), or (ii) the suspension time (10, 30, 60, 120, 240 min) at a fixed HgCl_2_ concentration (500 ppm). In a typical experiment, in a 15 mL plastic vial provided with a plug, i-Zn (∼20 mg, 9 × 10^–2^ mmol) was suspended in HgCl_2(aq)_ (2 mL) and kept under magnetic stirring for the selected time lapse. The solid was then separated from the mother liquor by centrifugation (10 min, 3500 rpm) and dried at room temperature under a flow of N_2_ for 4 h. Recovered mass vs initial i-Zn mass: 53–78%. All of the samples were analyzed by PXRD with the diffractometer described above (2θ range = 5.0–35.0°, step = 0.02°, time *per* step = 1 s, 0.2 mm deep aluminum sample holder). A PXRD qualitative analysis enabled us to identify the presence of the noninterpenetrated phase with clathrated HgCl_2_ (HgCl_2_@ni-Zn), together with the interpenetrated one, in all of the recovered samples. The presence of mercury and chlorine was assessed by X-ray fluorescence (see Section 2.1). On the samples with an appreciable amount of HgCl_2_@ni-Zn (100–5000 ppm), after an adequate PXRD data collection (2θ range = 5.0–105.0°, step = 0.02°, time per step = 10 s, 0.2 mm deep silicon free-background sample holder), a quantitative analysis was carried out. To this aim, the framework of the two MOFs was built based on the crystallographic information retrieved before (see Section 2.5), adopting idealized bond distances and angles for the BPE^2–^ ligand.^21^ Then, keeping the two frameworks fixed, the electronic density within the 1D channels of HgCl_2_@ni-Zn was modeled with the use of HgCl_2_·2H_2_O molecules,^28,29^ varying the position of their center of mass and their orientation with the Simulated Annealing approach, as well as their site occupation factor. The HgCl_2_·2H_2_O bond distances and angles were idealized^30^ starting from those retrieved from a geometry optimization (see Section 2.13). In modeling the HgCl_2_@ni-Zn system, taking advantage of the results of theoretical calculations (vide infra), we adopted a soft restrain involving the mercury atom and the carbon atoms of the triple bond. Indeed, unrestrained minimization resulted to be unfeasible due to the existence of a rather flat hypersurface with several relative minima. Hg^II^ vs Zn^II^ metal exchange in the MOF nodes was excluded upon letting the site occupation factor of the Zn^II^ cations refine. The crystal structure of HgCl_2_@ni-Zn and i-Zn was finally refined with the Rietveld method. The background and instrumental contribution to the peak profile were described as detailed in Section 2.5. The sample contribution to the anisotropic peak shape was accounted for by means of spherical harmonics. Figure S3 shows the Rietveld refinement plots of the 300 ppm/1 h and 500 ppm/2 h samples as representative examples, while Table S2 collects key information for all of the refinements.

### 2.11 Clathration Selectivity

The selectivity of the system under investigation toward HgCl_2_, a salt that does not ionize in water,^28^ was verified suspending powdered i-Zn (∼20 mg) in 1.8 × 10^–3^ M aqueous solutions (2 mL) of NaCl, KCl, MgCl_2_·6H_2_O or CaCl_2_·2H_2_O for 1 h [the same molar concentration of a 500 ppm solution of HgCl_2(aq)_ was adopted; see Table S3 for the analytes concentration in ppm]. The solid was recovered as described in Section 2.9 and analyzed by PXRD, acquiring the data in the 2θ range 3.0–35.0° with a step of 0.02° and a time *per* step of 1 s, and subsequently treating them with a whole powder pattern refinement with the Le Bail approach using TOPAS-R v.3.

### 2.12 Electronic-State Transition Spectroscopy Measurements

The solid-state UV–vis absorption spectra were acquired with a Jasco V-770 UV–vis–NIR spectrophotometer equipped with an ISN-923 60 mm integrating sphere and an explicitly designed solid-state sample holder. The fluorescence excitation and emission spectra were measured with a Jasco FP 8500 spectrofluorimeter. The powdered batches were introduced in the same sample holder used for the absorption measurements. The excitation beam was made to impinge the sample surface with an inclination angle of 36° (next to the theoretical magic angle), set through a goniometric lodge, to minimize the collection of excitation stray light. The instrument was interfaced with a dedicated acquisition software (Jasco Spectra Manager), which performs an online correction of the data with respect to the excitation lamp spectral radiance and the detector spectral quantum efficiency. The emission spectra were measured upon excitation at multiple wavelengths to investigate the fine structure of the absorption spectrum. The excitation spectra were collected fixing λ_obs_ to the main fluorescence emission peak (vide infra). The kinetics studies of the i-Zn-to-HgCl_2_@ni-Zn conversion induced by HgCl_2_ clathration were performed in suspension with a Fluorescence Master System fluorimeter (PTI) equipped with a magnetic stirrer: suspensions of i-Zn in distilled water at either 1 or 2 μg/mL concentration were prepared directly in the fluorimeter cuvette by letting the powder equilibrate in water under vigorous magnetic stirring for 30 min. Then, the cuvette was put in the fluorimeter holder, where the suspensions were kept under gentle magnetic stirring throughout the whole measurement to avoid precipitation. The desired concentration of HgCl_2(aq)_ was added and time-lapse fluorescence acquisition experiments were concomitantly started: the fluorescence emission intensity at (470 ± 4) nm was recorded every second at 90° to the excitation beam through a 400 nm long-wavelength pass filter (Corion, Holliston, MA). The excitation wavelength was set at (365 ± 4) nm. The data were acquired through the software Felix 2000.

Time-resolved fluorescence decay patterns were reconstructed by applying the time-correlated single-photon counting (TCSPC) technique, exploiting a system endowed with 30 ps temporal resolution, which is fully described elsewhere.^31^ The powders were deposed between two custom-made quartz windows (Crystran Limited, Poole, U.K.), sealed with parafilm, and held in the excitation laser beam (355 nm wavelength, 113 MHz repetition rate, 5.5 ps pulse duration) at the magic angle with the help of a rotator. Fluorescence was collected through the same 400 nm long-wavelength pass filter used in the kinetics experiments by means of a 20× microscope objective, and it was focused onto the sensitive area of a single-photon avalanche diode (SPAD, MicroPhoton Devices mod. SPCM, Bolzano, Italy). A suitable attenuation of the excitation beam by means of neutral density filters (Thorlabs) assured limitation of the fluorescence photocounts to <100 kHz, to achieve the single-photon detection regime. The current pulses corresponding to detected fluorescence photons were timed by means of a SPC150 TCSPC integrated PC board (Becker & Hickl GmbH, Berlin, GE). The STOP pulses were provided by a fast pin photodiode internal to the laser, triggered by a cavity loss. Ten fluorescence decay patterns were collected for each sample and fitted to double-exponential decays above a constant background by exploiting a hand-written Matlab routine based on a Levenberg Marquardt minimization algorithm. Addition of further decay components resulted in retrieval of the same value for two decay time constants, with no improvement of the fit quality. The reported values of the fitting parameters are averages over the 10 parallels, with errors corresponding to the pertaining standard deviations.

### 2.13 Electronic Structure Modeling

All of the electronic structure calculations were carried out at the B3LYP/6-31+g(d,p) level^32^ in the gas phase or employing a continuous solvent model PCM by means of the Gaussian09 suite of codes.^33^ The LANL2DZ basis set was instead used for mercury and chlorine atoms. Models for i-Zn and ni-Zn were built starting from the experimental crystal structures. A single ligand was used to describe the less crowded environment around the carbon–carbon triple bond in ni-Zn; three neighboring ligands were used to mimic the locally dense molecular environment around the same functional group in i-Zn. As the anionic nature of the ligands may be a key factor in defining both the spectroscopic properties of the two MOFs and the interactions involving the MOF pore walls, we deprotonated the pyrazole rings and neutralized their negative charge with lithium cations. The latter were chosen to minimize the computational cost involved in the electronic structure calculations while maintaining sufficiently strong acidic properties for the charge-neutralizing cations. The position of carbon and nitrogen atoms was kept unchanged in all calculations to maintain the disposition found in the experimental crystal structures; all of the other atoms were instead subject to structural optimization by minimizing the electronic energy with respect to their coordinates. All partially optimized structures were tested by computing energy second derivatives. The calculation of the vertical electronic excitations for the energy-minimized structures was conducted at the TD-DFT level using the same density functional theory/basis set combination employed for the structural optimization.

Two binding modalities between HgCl_2_ and the ligands were explored, namely, one with mercury directly facing the carbon–carbon triple bond, and another with a T-shaped structure featuring a chlorine pointing toward the center of the multiple bond. The latter was investigated as halogen atoms may present a reduced screening of their nuclear charge when involved in covalent bonds, the lower electron density being localized on the opposite side of the bond itself. Invariably, we found that the T-shape geometry was not maintained during the structural optimization.

## 3 Results and Discussion

### 3.1 Synthesis

**Scheme 2:**
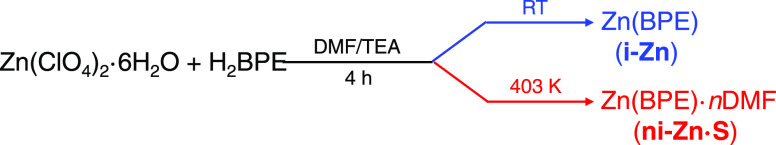
Synthesis of i-Zn and ni-Zn·S

The two MOFs ni-Zn·S and i-Zn can be selectively isolated in the form of off-white microcrystalline powders adopting the same reaction conditions except the reaction temperature (Scheme 2).

### 3.2 Characterization of the ni-Zn·S and i-Zn MOFs

#### 3.2.1 Crystal Structure Analysis

The noninterpenetrated polymorph Zn(BPE) (ni-Zn·S) can crystallize in both tetragonal (*P*4_2_/*mmc*) and orthorhombic (*Cccm*, a proper subgroup of *P*4_2_/*mmc*) space groups, depending on the guest molecules disposition.

**Figure 1:**
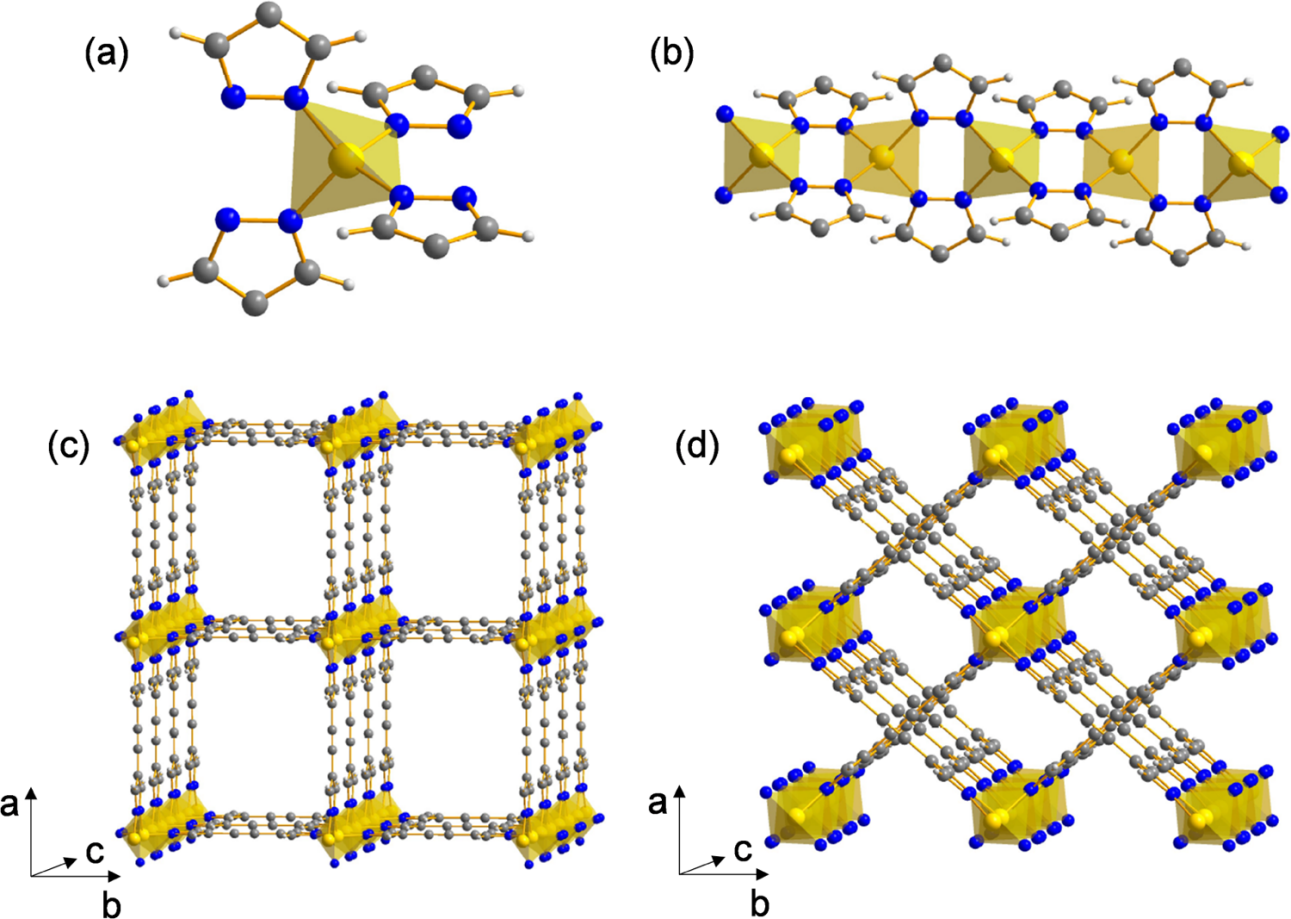
(a) Representation of the tetrahedral ZnN_4_ stereochemistry of the zinc(II) ion in the title MOFs. (b) The μ-pyrazolate coordination mode along the 1D chains running parallel to the [001] crystallographic direction in the title MOFs. (c) Portion of the crystal packing of ni-Zn·S viewed in perspective along the [001] crystallographic direction. The tetragonal modification (see Section 3.2.1) was arbitrarily chosen to draw the picture. The solvent molecules have been omitted for the sake of clarity. (d) Portion of the crystal packing of i-Zn viewed in perspective along the [001] crystallographic direction. Element color code: carbon, gray; hydrogen, light gray; nitrogen, blue; zinc, yellow. Main bond distances and angles in ni-Zn·S: Zn–N, 1.9076(2) Å; Zn···Zn, 3.6346(3) Å; N–Zn–N, 106.47(1)–110.99(1)°. Main bond distances and angles in i-Zn: Zn–N, 1.9673(1) Å; Zn···Zn, 3.6507(2) Å; N–Zn–N, 108.11(1)–111.78(1)°.

This occurrence has already been observed with other Zn(bipyrazolate) MOFs.^34^ Disregarding the different symmetry, which leads to the presence of square or rhombic channels, the structural motif is preserved. In the following, the description focuses on the tetragonal form. ni-Zn·S is isoreticular to Zn(BPZ).^18^ Hence, in its crystal structure tetrahedral ZnN_4_ nodes (Figure 1a; see the figure caption for the main bond distances and angles at the metal ion) and exo-tetradentate ligands (Figure 1b) form a 3D (4,4)-connected network (Figure 1c) of PtS topology. The network displays 1D channels ∼0.8 × 0.8 nm^2^ wide,^35^ which run along the [001] crystallographic direction and are occupied by disordered DMF molecules. At room temperature and pressure conditions, neglecting the solvent molecules, the empty volume amounts to ∼66%.^36^

The interpenetrated^37^ Zn(BPE) MOF (i-Zn) crystallizes in the orthorhombic space group *Pccm*.

The crystal structure is composed of a 2-fold interpenetrated (Figure S4) 3D (4,4)-connected network of PtS topology, formed by tetrahedral ZnN_4_ nodes and exo-tetradentate spacers (Figure 1a,b; see the figure caption for the main bond distances and angles at the metal ion), as in the case of the noninterpenetrated phase. Interpenetration occurs about the ligand triple bonds (Figure 1d); the value of the centroid-to-centroid distance [3.6507(2) Å] suggests the insurgence of π–π interactions. Network interpenetration at the ligand carbon–carbon triple bonds has been already observed in the past, e.g., in Zn(BPEB) [H_2_BPEB = 1,4-bis(1*H*-pyrazol-4-ylethynyl)benzene].^38^ Despite interpenetration, 1D channels ∼0.4 × 0.4 nm^2^ wide run along the [001] crystallographic direction (Figure 1d). The empty volume is ∼22%, significantly lower than that of ni-Zn.

#### 3.2.2 Thermal Behavior

The variable-temperature PXRD experiments highlighted that ni-Zn and i-Zn are stable up to 723 K and 763 K, respectively. Further details on the thermal behavior of the ligand and the two MOFs are reported in Section S4 (Figures S5–S7), to which the reader is referred.

#### 3.2.3 Textural Properties

**Figure 2:**
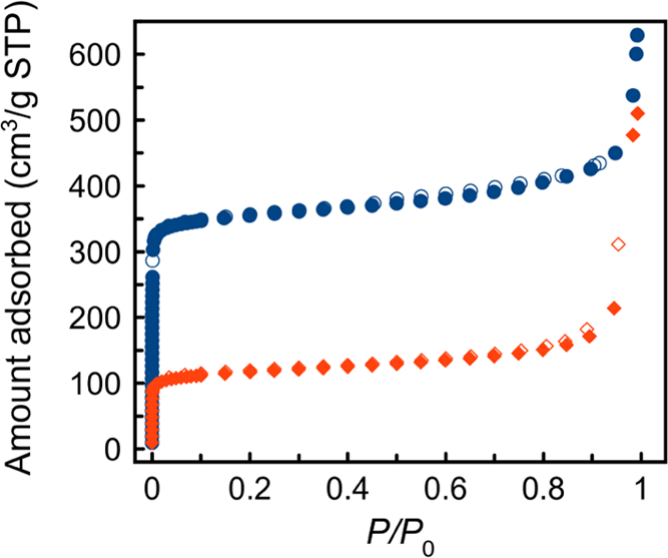
Comparison between the N_2_ adsorption isotherms at 77 K of ni-Zn (blue circles) and i-Zn (red diamonds). Empty symbols depict the desorption branch.

The permanent porosity of ni-Zn and i-Zn was evaluated by acquiring volumetric N_2_ adsorption isotherms at 77 K after thermal activation (see Section 2 and Figure S8). ni-Zn and i-Zn show type I adsorption isotherms, characteristic of microporous materials, and adsorb different amounts of N_2_ (Figure 2). Interestingly, the calculated Brunauer–Emmett–Teller (BET) specific surface areas of the two MOFs show the same trend of the empty volume estimated from the crystal structures (see above): the BET specific surface area of ni-Zn is nearly the triple that of i-Zn, 1380 and 442 m^2^/g, respectively.^39^

#### 3.2.4 Water Stability

After a 24-h suspension, ni-Zn is completely transformed into i-Zn (Figure S10). In accordance with this transformation, the BET specific surface area of the recovered material, retrieved from the N_2_ adsorption isotherm acquired at 77 K (Figure S8), amounts to 440 m^2^/g, as that of as-synthesized, activated i-Zn (see above). On the other hand, i-Zn is stable in water for at least 15 days, without substantial changes in its PXRD pattern (Figure S11).

#### 3.2.5 Theoretical Modeling

Figure S12 shows the optimized structure of the two model systems employed to represent i-Zn and ni-Zn, as detailed in Section 2.13. These optimized structures were exploited to both interpret the electronic-state transition spectroscopic features of the two MOFs and restrain the PXRD modeling of the HgCl_2_@ni-Zn system (vide infra).

#### 3.2.6 Spectroscopic Characterization of the Electronic-State Transitions

In view of testing the suitability of i-Zn as a luminescent sensor of Hg-containing pollutants in water, the optical spectroscopic properties of i-Zn and ni-Zn·S were preliminarily assessed. To this aim, the solid-state UV–vis absorption spectra of the H_2_BPE ligand and of the i-Zn and ni-Zn·S MOFs were recorded. Furthermore, in-depth steady-state fluorescence emission and excitation spectroscopy studies were performed on the same specimens, and time-resolved fluorescence experiments were undertaken. This characterization, reported in Section S8 (Figures S13–S16), enabled us to evidence, in the excitation spectra of both MOFs, a band peaked at 365 nm (Figure S15a). The theoretical modeling of the two MOFs according to the simplified systems for the noninterpenetrated and the interpenetrated frameworks (Figure S12) offers valuable clues to evince the nature of this band. Indeed, both models suggest the presence of a few electronic transitions with a high oscillator strength around 389–390 nm. These transitions are characterized by a charge transfer from the molecular HOMO to empty orbitals localized on the cations (in the noninterpenetrated framework), or from a linear combination of the three occupied molecular orbitals with the highest eigenvalues to empty orbitals localized on the cations (in the interpenetrated framework). Even though the theoretically estimated energy gap somewhat underestimates the experimental one due to the known shortcomings of hybrid functionals,^40^ this finding supports the attribution of the observed UVA transition to a charge transfer process. Upon excitation within this band, both i-Zn and ni-Zn·S emit bluish fluorescence. The emission spectral line shape (Figure S15b) is very similar for the two MOFs, and it is characterized by a double-peaked band with relative maxima at ∼450 and ∼470 nm. However, the fluorescence emission intensity is notably different. Indeed, the band is rather intense for ni-Zn·S, while it is substantially quenched in the case of i-Zn. We interpret this occurrence as due to the π–π stacking interactions among the triple bonds of neighboring ligands, as highlighted in Section 3.2.1.

The fluorescence intensity emitted at 470 nm upon excitation at 365 nm was adopted as the spectroscopic indicator of the i-Zn-to-HgCl_2_@ni-Zn conversion advancement in the studies devoted to luminescence-based HgCl_2_ sensing (vide infra).

### 3.3 Interaction with HgCl_2_

#### 3.3.1 Characterization of the i-Zn-to-HgCl_2_@ni-Zn Conversion in HgCl_2(aq)_

When i-Zn (∼20 mg, 9 × 10^–2^ mmol) was suspended in HgCl_2(aq)_ aqueous solutions (2 mL) of increasing concentration (in the 10–5000 ppm range, see Section 2.10 and Table S1) for 1 h, we observed partial conversion to HgCl_2_@ni-Zn with no appreciable crystal size changes for the residual i-Zn (Figure S17): the relative abundance of HgCl_2_@ni-Zn over i-Zn progressively increased with the salt concentration.

**Figure 3:**
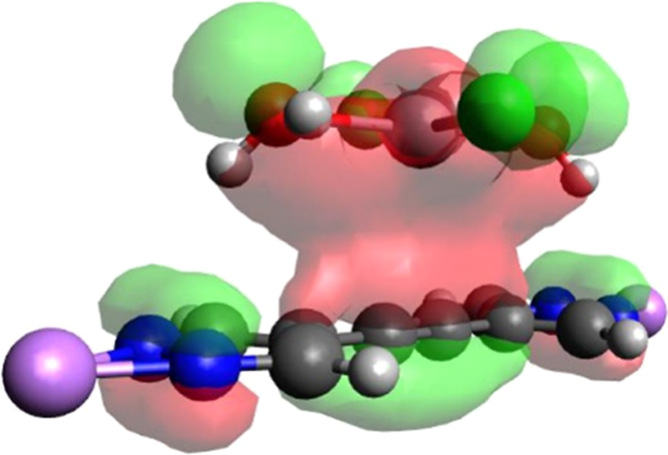
Optimized geometrical structure for the dihydrated HgCl_2_ molecule interacting with the BPE^2–^ triple bond. The molecular orbital involved in the donation of electron density from the triple bond to Hg^II^ is also shown. Atoms color code: carbon, gray; hydrogen, light gray; chlorine, green; lithium, violet; mercury, dark gray; nitrogen, blue; oxygen, red.

To shed some light on the molecular bases of this peculiar behavior, we first undertook theoretical calculations aimed at investigating the possibility of preferential interactions among HgCl_2_ and the BPE^2–^ linker. It is worth recalling at this stage that HgCl_2_ behaves as a nonelectrolyte in water,^28^ an aspect which was typically neglected by the works dedicated to MOFs adsorption of Hg^II^ from water. Accordingly, we initially added molecular HgCl_2_ either close to a carbon–carbon triple bond (the one on the central ligand in the model of the interpenetrated framework; Figure S12) or over a pyrazolate ring. In the case of the model of the interpenetrated framework, the HgCl_2_ moiety systematically displaced from any of the initial locations to coordinate, sitting in the same plane of the ring, with a pyrazolate nitrogen atom. While a similar system was also obtained with the noninterpenetrated model starting the optimization with the HgCl_2_ molecule over the heteroaromatic ring, placing HgCl_2_ close to the triple bond provided a stable η_2_-coordinated species (see Figure 3) with a binding energy of ∼32.6 kJ/mol. At a second stage, a more refined model was implemented to unravel the details of the HgCl_2_ docking to the BPE^2–^ ligands, which took into account that the metal atom of this salt is known to coordinate few water molecules despite its molecular nature.^29^ In agreement with ref (29), we thus decided to model HgCl_2_ as coordinating three water molecules while in solution. Despite this, we found that the most stable species obtained following the coordination of mercury to the triple bond (affording a binding energy of ∼46.0 kJ/mol) contained two water molecules bound to a square planar Hg^II^ ion, i.e., with a water molecule in the original solvation shell being substituted by the ligand triple bond upon coordination (Figure 3).^41^ In conclusion, the small but negative energy retrieved for the HgCl_2_ coordination to the ni-Zn model structure, but not to the i-Zn model structure, de facto provides a possible driving force for the structural changes from i-Zn to HgCl_2_@ni-Zn evidenced experimentally.

The i-Zn to HgCl_2_@ni-Zn transformation, consisting in the formation of a noninterpenetrated framework starting from an interpenetrated one, implies Zn–N bonds rupture and subsequent reformation.

Robust experimental evidence can be brought in support of such interconversion dynamics. Indeed, as detailed in Section 2.10, the solid is recovered from the suspension with a yield lower than expected, suggesting that i-Zn is dissolved, and it is only partially recovered as HgCl_2_@ni-Zn. Indeed, zinc is invariably detected in the mother liquors by XRF (Figure S18). Moreover, the ^1^H NMR spectrum of the mother liquors recovered after 1 h suspension of i-Zn in a 500 ppm D_2_O solution of HgCl_2_ shows a signal at 8.09 ppm, which can be attributed to the hydrogen atoms of the pyrazole heteroaromatic ring (Figure S19). Finally, in all of the cases, X-ray fluorescence enabled us to assess the presence of mercury and chlorine in the recovered samples (Figure S20), suggesting that the new ni-Zn phase builds up around the salt molecules. These observations confirm that a dynamic equilibrium is in action between the two MOFs and their solubilized building blocks, and thereby suggest that the bond rupture and reformation are eased by i-Zn dissolution. Another relevant result of the mother liquor analyses is that in all of the tested specimens, ICP-MS demonstrated the quantitative removal of mercury, with only trace levels left (0.003–1.2 ppm).

#### 3.3.2 Quantitative Assessments on the [HgCl_2_] Dependence of the i-Zn-to-HgCl_2_@ni-Zn Equilibrium

**Figure 4:**
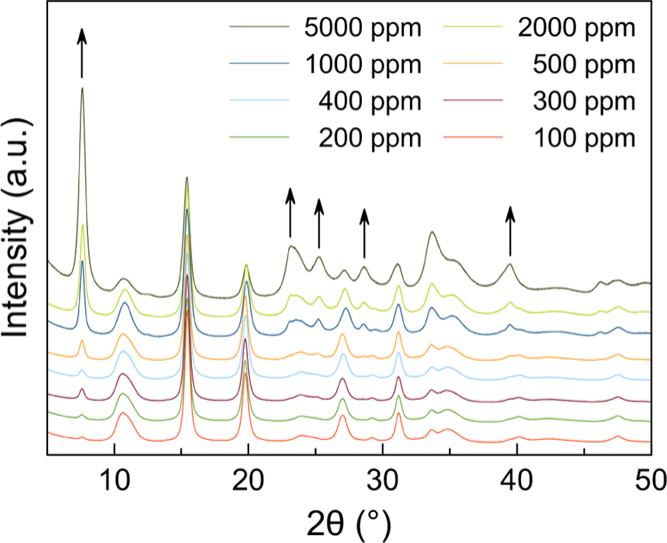
Low-to medium-angle portion of the powder X-ray diffraction patterns of the HgCl_2_@ni-Zn/i-Zn samples recovered after 1 h suspension of i-Zn in HgCl_2_ aqueous solutions of concentration in the range 100–5000 ppm. The peaks belonging exclusively to HgCl_2_@ni-Zn are indicated with arrows. For the entire PXRD patterns also of the 10, 50, and 75 ppm suspensions, the reader is referred to Figure S17.

Treatment of the PXRD data of the samples recovered from the 100–5000 ppm solutions^42^ (Figure 4) was performed to confirm the presence of the HgCl_2_·2H_2_O guest molecules and locate them within the channels.^43^ A preliminary exploration of the HgCl_2_@ni-Zn system highlighted the existence of a rather flat hypersurface with several relative minima: unconstrained modeling resulted in comparably accurate descriptions of the observed PXRD pattern with the HgCl_2_·2H_2_O guest interacting with the triple bond through either the metal or the halogen atom. It is known that chlorine is endowed with an exceptionally high quadrupole moment. To rule out quadrupolar interactions as the driving force leading to HgCl_2_@ni-Zn formation, a combined theoretical and experimental approach was adopted.

As to theoretical calculations, additional structural optimizations for the model of ni-Zn were carried out with HgCl_2_·2H_2_O in a T-shape geometry with respect to the ligand triple bond, a chlorine atom pointing directly toward the latter to maximize the electrostatic interaction. In the end, however, the initial geometry was not maintained.

In parallel, the interaction of i-Zn with a selection of chloride salts with metals (M) of the first and second groups (M = Na, K, Mg, Ca) was probed by preparing suspensions in experimental conditions similar to those used with HgCl_2(aq)_. In no cases, after 1 h suspension, the (M^I/II^,Cl^–^)@ni-Zn species was formed: as verified by PXRD, the recovered precipitate only contained the pristine i-Zn phase (Figure S21). Furthermore, XRF spectra of the retrieved powders confirmed the absence of the scrutinized cations. Besides corroborating the in silico results, pointing toward the exclusion of a specific interaction of chlorine with the ligand triple bonds in HgCl_2_@ni-Zn, these data may be considered as a preliminary test of the selectivity of i-Zn toward salts not dissociating in water, as HgCl_2_.

**Figure 5:**
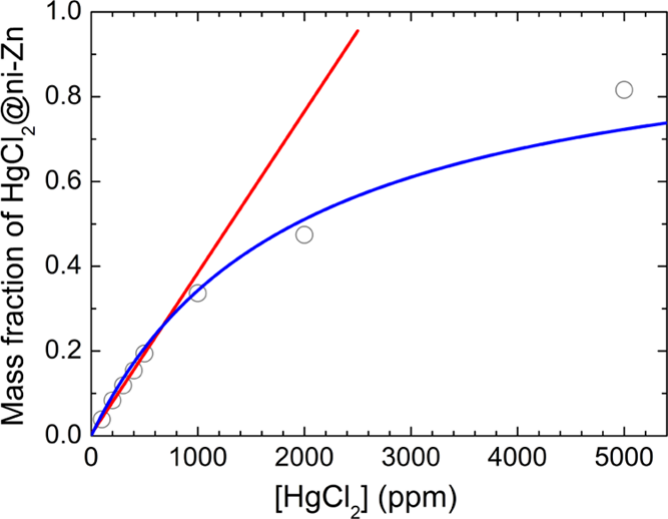
Mass fraction of HgCl_2_@ni-Zn formed as a function of the concentration of the HgCl_2_ aqueous solution. The red line represents the best linear fit of the data in the range of salt concentrations [HgCl_2_] ≤ 500 ppm. The blue line represents the best fit of the whole dataset to a Langmuir model.^44^

Based on the above results, a soft restrain involving the mercury atom and the carbon atoms of the triple bond was imposed to treat the PXRD data. This enabled us to retrieve the mass percentage of HgCl_2_@ni-Zn formed (Figure 5 and Table S2) as a function of [HgCl_2_]. Although the percentage of HgCl_2_@ni-Zn obviously tends to saturate at high [HgCl_2_],^44^ the two quantities are, with optimal approximation, directly proportional in the salt concentration range in which i-Zn is initially in large molar excess with respect to HgCl_2_ ([HgCl_2_] ≤ 500 ppm, see Table S1). As for the location of the guest, the HgCl_2_ molecules are disordered about a 2/*m* crystallographic position (Figure S22 and Table S2). The shortest HgCl_2_···linker nonbonding interactions involve the triple bond carbon atoms [Hg···C_triple bond_ = 3.098(3)–3.335(2) Å, Cl···C_triple bond_ = 3.045(4)–3.735(5) Å]. The absence of stronger host-guest interactions disrupting the ligand symmetry is witnessed also by IR spectroscopy: the IR spectrum of the HgCl_2_@ni-Zn/i-Zn mixture is superimposable to those of ni-Zn and i-Zn (Figure S1).

**Figure 6:**
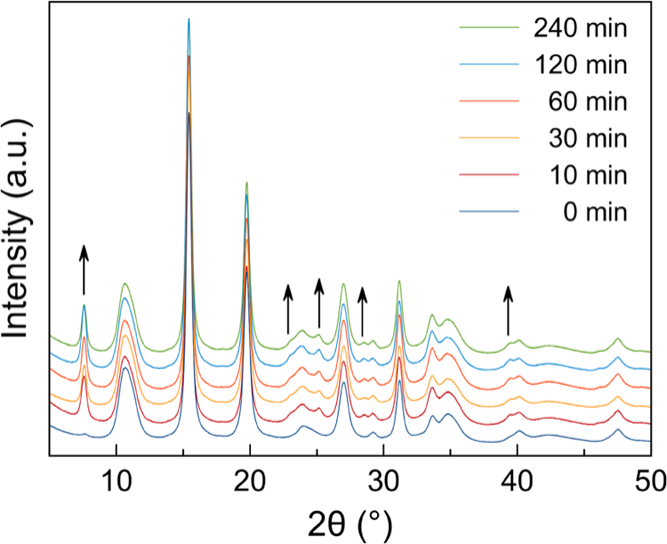
Low-to medium-angle portion of the powder X-ray diffraction patterns of the HgCl_2_@ni-Zn/i-Zn samples recovered after suspension of i-Zn in 500 ppm HgCl_2_ aqueous solutions for different time lapses. The peaks belonging exclusively to HgCl_2_@ni-Zn are highlighted with an arrow. For the entire PXRD patterns, the reader is referred to Figure S23.

In a different experiment, equal-weight samples of i-Zn (20 mg, 9 × 10^–2^ mmol) were suspended in equal-volume aliquots of 500 ppm HgCl_2(aq)_ for different time lapses (10–240 min; Figure 6). As proved by PXRD (Table S2), in these conditions the equilibrium between the HgCl_2_@ni-Zn and i-Zn phases is reached within 10 min, with no appreciable crystal size changes for the residual i-Zn or for the growing ni-Zn.

#### 3.3.3 Fluorescence-Based Sensing of HgCl_2_

**Figure 7:**
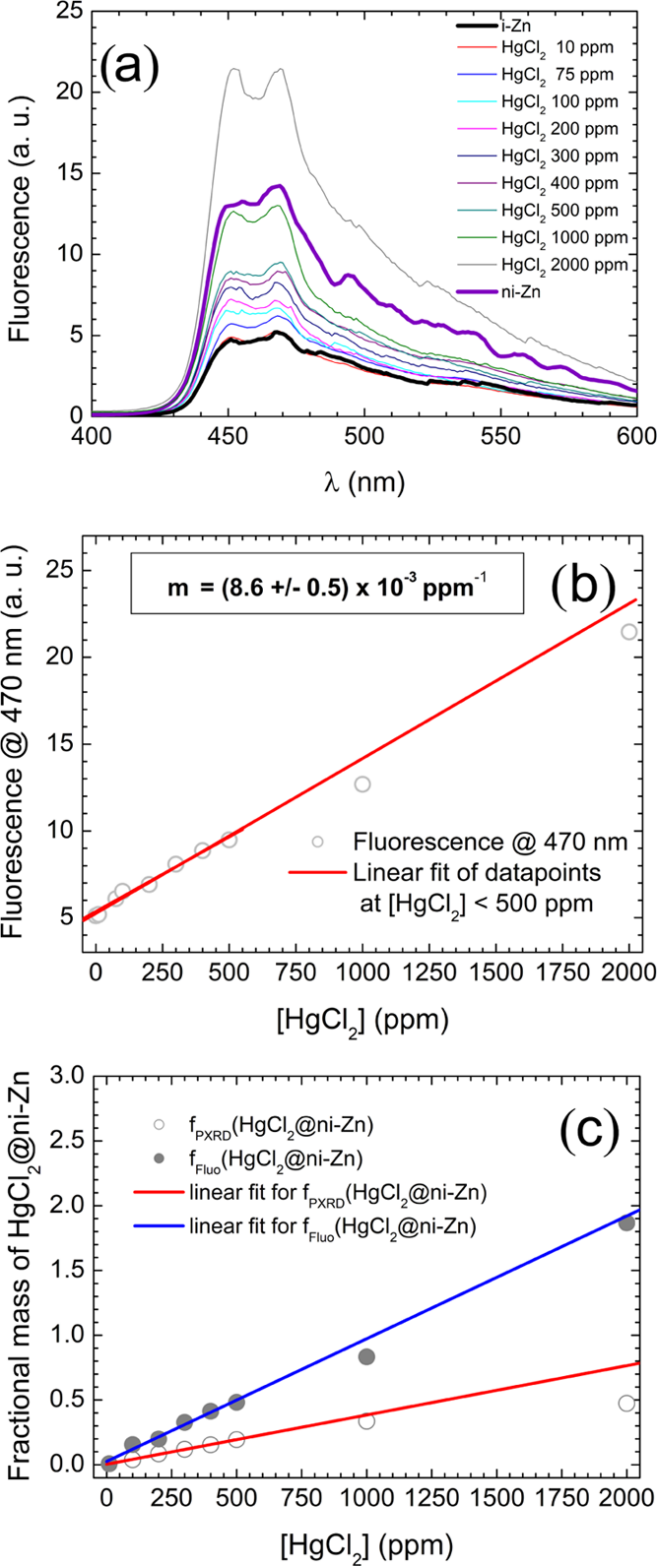
(a) Fluorescence spectra, recorded upon excitation at 365 nm, of the Hg@ni-Zn/i-Zn samples obtained at various HgCl_2 (aq)_ concentrations and previously investigated by PXRD. The thicker lines represent the emission of pristine i-Zn (black) and ni-Zn·S (violet), respectively. (b) Plot of the fluorescence value measured at 470 nm vs HgCl_2(aq)_ concentration. The red line represents the best linear fit of the experimental data in the range [HgCl_2_] ≤ 500 ppm; the resulting slope is reported.^45^ (c) *f*_Fluo_(HgCl_2_@ni-Zn) and *f*_PXRD_(HgCl_2_@ni-Zn) vs [HgCl_2_] [see the main text for *f*_Fluo_(HgCl_2_@ni-Zn) and *f*_PXRD_(HgCl_2_@ni-Zn) definition]. The red and blue lines represent the best fit of the data for [HgCl_2_] ≤ 500 ppm.

The fluorescence emission of the HgCl_2_@ni-Zn/i-Zn samples obtained at different HgCl_2(aq)_ concentrations and previously characterized through PXRD (see Section 3.3.1) was measured upon excitation at 365 nm.^45^ The pertinent spectra are plotted in Figure 7a. The spectral line shape is not significantly modified by HgCl_2(aq)_ clathration. At variance, a systematic increase in the emission intensity is observed, which results roughly proportional to the HgCl_2(aq)_ concentration, as shown in Figure 7b. Based on the PXRD observations, we interpret the increasing trend of fluorescence intensity at incrementing [HgCl_2_] as the result of the progressive conversion of i-Zn into the highly fluorescent HgCl_2_@ni-Zn species, fostered by the clathration of HgCl_2_·2H_2_O molecules. However, at high [HgCl_2_] the fluorescence of the HgCl_2_@ni-Zn/i-Zn mixture exceeds that of pure as-synthesized ni-Zn·S. This result may be explained by hypothesizing an increase in the ni-Zn fluorescence quantum yield upon clathration of HgCl_2_. Indeed, the exceptional efficacy of mercury in promoting spin–orbit coupling interactions is well known.^46^

The slope of the fitting line yields an estimate of the ultimate sensitivity of the luminescence assay to HgCl_2(aq)_ in the present experimental conditions. Namely, since the slope is on the order of one-hundredth of fluorescence arbitrary units per ppm, the minimum HgCl_2(aq)_ concentration detectable on solid-state samples should be on the order of 10 ppm, corresponding to 2% of the i-Zn signal. Indeed, measuring the spectrum of an appositely prepared sample suspended for 1 h in 10 ppm HgCl_2(aq)_ we obtained a fluorescence value, at 470 nm, slightly higher than that of i-Zn (see the corresponding data point in Figure 7b). These fluorescence data may be in principle exploited to set up a luminescence-based mercury quantitation assay allowing us to determine the amount of HgCl_2(aq)_ in the [HgCl_2_] range not exceeding 500 ppm, i.e., when i-Zn is initially in large molar excess with respect to HgCl_2_ and the fluorescence intensity results to be linear with [HgCl_2_].

Namely, if 20 mg of i-Zn are suspended into 2 mL of an aqueous solution of unknown HgCl_2_ concentration, left under stirring for 1 h, and then dried applying the same procedure described in Section 2.9, and if the fluorescence of the obtained powder is measured with the same fluorimeter settings used to obtain Figure 7, the concentration of HgCl_2_ may be straightforwardly determined as
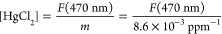
1where *F*(470 nm) = *f*(i-Zn) × *F*(i-Zn) + *f*(HgCl_2_@ni-Zn) × *F*(HgCl_2_@ni-Zn) is the fluorescence intensity detected at 470 nm, function of the fluorescence *F* of i-Zn and HgCl_2_@ni-Zn and of their mass ratio *f*, and *m* is the slope of the best-fitting line in Figure 7b.

Moreover, if the ni-Zn fluorescence quantum yield were not affected by HgCl_2_ clathration, the molar fraction *f* of HgCl_2_@ni-Zn could be expressed as
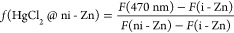
2Figure 7c compares the mass fractions of HgCl_2_@ni-Zn derived from the rough data plotted in Figure 7b applying eq 2 with the fractions of HgCl_2_@ni-Zn retrieved by PXRD (reported from Figure 5). A linear fit of the two plots in the ≤ 500 ppm salt concentration range was used to derive a calibration curve to extract the mass fraction of HgCl_2_@ni-Zn as a function of the fluorescence intensity measured at 470 nm for a sample suspended in a solution of HgCl_2(aq)_ of unknown concentration
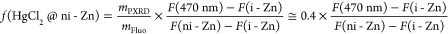
3where *m*_PXRD_ = (3.8 ± 0.1) × 10^–4^ ppm^–1^ is the slope of the best linear regression interpolating the PXRD data (red line in Figure 5, also reported in Figure 7c for the sake of comparison) and *m*_Fluo_ = (9.5 ± 0.7) × 10^–4^ ppm^–1^ is the slope of the best linear regression interpolating the fluorescence data (blue line in Figure 7c).^47^

**Figure 8:**
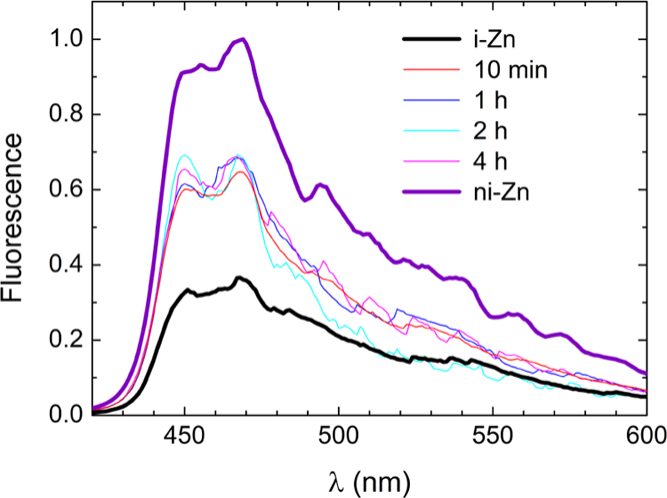
Fluorescence spectra recorded upon excitation at 365 nm of the HgCl_2_@ni-Zn/i-Zn samples obtained upon suspension of i-Zn in 500 ppm HgCl_2 (aq)_ for different time lapses and previously investigated by PXRD. The thicker lines represent the emission of the pristine i-Zn (black) and ni-Zn·S (violet) batches, respectively.

The samples exposed to 500 ppm HgCl_2(aq)_ for different time lapses were also analyzed by fluorescence spectroscopy. Results compatible with those yielded by PXRD (see Figure 6) were obtained. Indeed, the emission spectra of all of the samples, plotted in Figure 8, are equal within the experimental errors, indicating that the equilibrium between i-Zn and HgCl_2_@ni-Zn is reached within the first 10 min of suspension.

**Figure 9:**
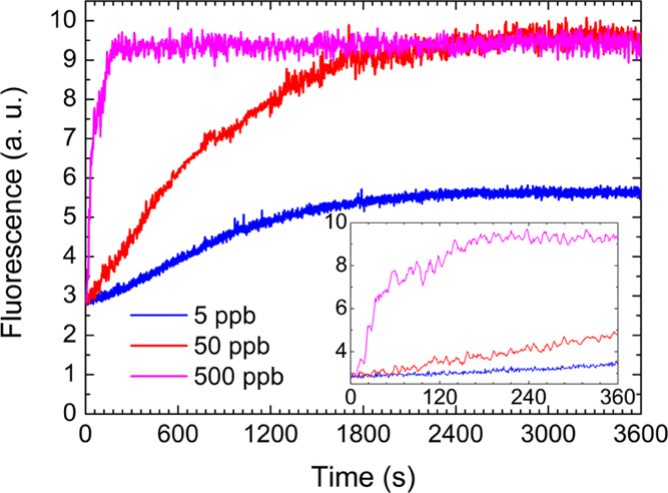
Fluorescence at 470 nm of 1 μg/mL suspensions of i-Zn in HgCl_2 (aq)_ solutions at the indicated concentrations as a function of suspension time. The data suggest progressive conversion of i-Zn to HgCl_2_@ni-Zn. The fast conversion dynamics in the case of 500 ppb HgCl_2 (aq)_ can be appreciated in the magnification of the first 6 min displayed in the inset. Worthy of note, the 50 ppb solution preserves the same MOF-to-HgCl_2 (aq)_ concentration ratio as the 500 ppm solution used to obtain the solid-state samples.

Finally, a proof of principle test of the ability of i-Zn to reveal the presence of Hg-containing pollutants in water was undertaken. First, a panel of progressively diluted suspensions in distilled water of as-synthesized i-Zn and HgCl_2_@ni-Zn/i-Zn^48^ were prepared. As shown in Figure S24, even at such a low concentration as 1 μg/mL the i-Zn fluorescence emission in water is easily and neatly detected, and the fluorescence increase consequent to HgCl_2_-mediated partial conversion to HgCl_2_@ni-Zn apparent. As a further step, we tried to monitor in real time the i-Zn-to-HgCl_2_@ni-Zn conversion adding HgCl_2(aq)_ at different concentrations to 1 μg/mL suspensions of i-Zn and recording the fluorescence intensity at 470 nm over time (see Section 2.12 for details). In Figure 9, we report the results obtained upon addition of 5, 50, and 500 ppb HgCl_2(aq)_ (blue, red, and magenta lines, respectively).^49^ The first two traces reach a plateau (corresponding to the achievement of dynamic equilibrium between i-Zn and HgCl_2_@ni-Zn) within ∼20 min. In these instances, the rate and equilibrium controlling factor seem to be the HgCl_2(aq)_ concentration, as the fluorescence increase is roughly 3-fold higher when the salt concentration is incremented from 5 to 50 ppb. However, the nonlinear scaling of the plateau fluorescence value with the HgCl_2(aq)_ concentration suggests that already at 50 ppb the transformation takes place in excess of salt^50^ with respect to solid i-Zn. This conclusion is confirmed by the fact that, increasing by another order of magnitude the salt concentration, the same plateau fluorescence value is reached (i.e., the same amount of HgCl_2_@ni-Zn is formed), although in a much shorter time. In this concentration range, the reaction seems to behave as a collision-limited process, as the interconversion time scales roughly linearly with the salt concentration.

This speculation is further supported by the data reported in Figure S25, in which the experiment is repeated at the same salt concentration (500 ppb) but doubling the i-Zn concentration to 2 μg/mL. The fluorescence plateau is reached about twice as quickly.^51^

From a barely applicative standpoint, the above results suggest that, at least if fluorescence is detected with a fluorimeter with technical specifications similar to those of our equipment, the detection limit for Hg-containing pollutants in water by means of a putative luminescence assay based on the i-Zn conversion to HgCl_2_@ni-Zn should be in the range of few ppb. Accordingly, the system promises to be adequate to assess the drinkability of waters according to the standards in force.

## 4 Conclusions

In this paper, relying on a multitechnique approach combining in silico modeling with advanced experimental techniques including powder X-ray diffraction and electronic-state transition spectroscopy, we characterized at the molecular level a peculiar transition between the interpenetrated i-Zn and noninterpenetrated ni-Zn MOFs prompted by suspension of the former in aqueous HgCl_2_. Indeed, through self-assembly around the mercury salt of the solubilized ligand and Zn^II^ ions, in equilibrium with solid i-Zn, HgCl_2_@ni-Zn is formed. Since both MOFs emit fluorescence with different quantum yields, the above transition can be exploited for HgCl_2_ sensing and quantification purposes. Remarkably, to the best of our knowledge, this is the first time that disruption of a water-stable MOF and concomitant self-assembly of a different one seeded by a heavy metal salt is observed. Starting from this previously unobserved mechanism, new-conception methods might be envisaged to pursue water assessment and purification.

## Supporting Information Available

Synthesis and analytical characterization of the H_2_BPE ligand (Section S1); IR spectra of ni-Zn, i-Zn, and HgCl_2_@ni-Zn (Section S2, Figure S1); powder X-ray diffraction structural characterization (Section S3); Rietveld refinement plots for ni-Zn·S, i-Zn, and t-i-Zn (Figure S2) and selected HgCl_2_@ni-Zn/i-Zn mixtures (Figure S3); details on the suspensions of i-Zn in HgCl_2_ aqueous solutions (Table S1); details of the Rietveld refinements on the PXRD data of the HgCl_2_@ni-Zn/i-Zn mixtures (Table S2); details of the alkaline and earth-alkaline metal chloride solutions prepared (Table S3); graphical representation of interpenetration in i-Zn (Figure S4); details on the thermal behavior of H_2_BPE, ni-Zn, and i-Zn (Section S4); TGA and DSC of H_2_BPE (Figure S5); thermal behavior of ni-Zn·S and i-Zn (Figures S6 and S7); textural properties (Section S5); pore size distribution of ni-Zn and i-Zn (Figure S8); PXRD patterns of ni-Zn and i-Zn collected pre- and post-adsorption isotherms (Figure S9); water stability of ni-Zn·S and i-Zn (Section S6, Figures S10 and S11); models for i-Zn and ni-Zn adopted for the theoretical calculations (Section S7, Figure S12); details on electronic-state transition spectroscopic characterization of H_2_BPE, ni-Zn, and i-Zn (Section S8); UV–vis absorption spectra of H_2_BPE, ni-Zn·S, and i-Zn (Figure S13); fluorescence emission spectra of H_2_BPE, ni-Zn, and i-Zn upon excitation in the UVB (Figure S14); fluorescence excitation spectra of ni-Zn and i-Zn and emission spectra upon excitation at the charge transfer band peak (Figure S15); fitting parameters retrieved from a fit of the experimental fluorescence decay patterns of ni-Zn, i-Zn, and an exemplary HgCl_2_@ni-Zn/i-Zn mixture (Table S4); fluorescence decay patterns of ni-Zn·S, i-Zn, and an exemplary HgCl_2_@ni-Zn/i-Zn mixture (Figure S16); details on HgCl_2_ adsorption (Section S9); PXRD patterns of the samples recovered after i-Zn suspension in HgCl_2 (aq)_ (Figure S17); XRF spectrum of selected mother liquors post i-Zn suspension in HgCl_2(aq)_ (Figure S18); ^1^H NMR spectrum of the mother liquors of the suspension of i-Zn in a D_2_O solution of HgCl_2_ (Figure S19); XRF spectrum of a HgCl_2_@ni-Zn/i-Zn mixture (Figure S20); PXRD evidence of i-Zn selectivity (Figure S21); position of the HgCl_2_ molecules in the 1D channels of HgCl_2_@ni-Zn (Figure S22); PXRD patterns of the suspension of i-Zn in HgCl_2(aq)_ 500 ppm for different time lapses (Figure S23); luminescence sensing of HgCl_2_ in water (Section S10); fluorescence spectra of 1 μg/mL suspension of as-synthesized i-Zn and of the HgCl_2_@ni-Zn/i-Zn system obtained from 500 ppm HgCl_2 (aq)_ for 1 h (Figure S24); and fluorescence at 470 nm of suspensions of i-Zn in HgCl_2(aq)_ solutions vs suspension time (Figure S25) (PDF)
