# Supplementary material for: Mercury Clathration-Driven Phase Transition in a Luminescent Bipyrazolate Metal–Organic Framework: A Multitechnique Investigation
Source: Chem Mater. 2023 Mar 22;35(7):2892–903. doi: 10.1021/acs.chemmater.2c03801 (PMC10100537; doi:10.1021/acs.chemmater.2c03801)
Supplement: Supplementary file 1 — cm2c03801_si_001.pdf [file cm2c03801_si_001.pdf]

Supplementary Information for

Mercury clathration-driven phase transition in a luminescent bipyrazolate metal-organic framework: a multi-technique investigation

Marco Moroni,[a] Luca Nardo[a,*], Angelo Maspero[a], Guglielmo Vesco[a], Marco Lamperti[a], Luca Scapinello[a], Rebecca Vismara[b], Jorge A.R. Navarro[b], Damiano Monticelli[a], Andrea Penoni[a], Massimo Mella[a], Simona Galli[a,c*]

[a] Dipartimento di Scienza e alta Tecnologia, Università degli Studi dell'Insubria, Via Valleggio 11, 22100 Como, Italy.

[b] Departamento de Química Inorgánica, Universidad de Granada, Av. Fuentenueva S/N, 18071 Granada, Spain.

[c] Consorzio Interuniversitario Nazionale per la Scienza e Tecnologia dei Materiali, Via Giusti 9, 50121 Firenze, Italy

Corresponding authors: simona.galli@uninsubria.it, luca.nardo@uninsubria.it

S1. Synthesis of the 1,2-bis(1*H*-pyrazol-4-yl)ethyne ligand S3

S1.1. Synthesis of 1-(1-ethoxyethyl)-4-iodo-1*H*-pyrazole, 1 S3

S1.2. Synthesis of 1-(1-ethoxyethyl)-4-((trimethylsilyl)ethynyl)-1*H*-pyrazole, 2 S3

S1.3. Synthesis of 1-(1-ethoxyethyl)-4-ethynyl-1*H*-pyrazole, 3 S4

S1.4. Synthesis of 1,2-bis(1-(1-ethoxyethyl)-1*H*-pyrazol-4-yl)ethyne, 4 S4

S1.5. Synthesis of 1,2-Bis(1*H*-pyrazol-4-yl)ethyne, H_2BPE S5

S2. IR spectroscopy S6

S3. Powder X-ray diffraction structural characterization S7

S4. Thermal behaviour S12

S5. Textural properties S14

S6. Water stability S16

S7. Theoretical calculations S17

S8. Electronic-state transition spectroscopy S18

S9. $HgCl_2$ adsorption S23

S10. Luminescence sensing of $HgCl_2$ S27

S11. References S28

S1. Synthesis of the 1,2-bis(1*H*-pyrazol-4-yl)ethyne ligand

The 1,2-bis(1*H*-pyrazol-4-yl)ethyne ligand (H_2BPE) was prepared with a five-step synthetic approach starting from the commercially available 4-iodopyrazole and through a double Sonogashira synthetic protocol (Scheme S1). An *N*-protection reaction was preliminarily carried out using ethyl-vinyl ether as protecting group. The complete synthetic protocol is detailed in the following, with reference to Scheme S1.

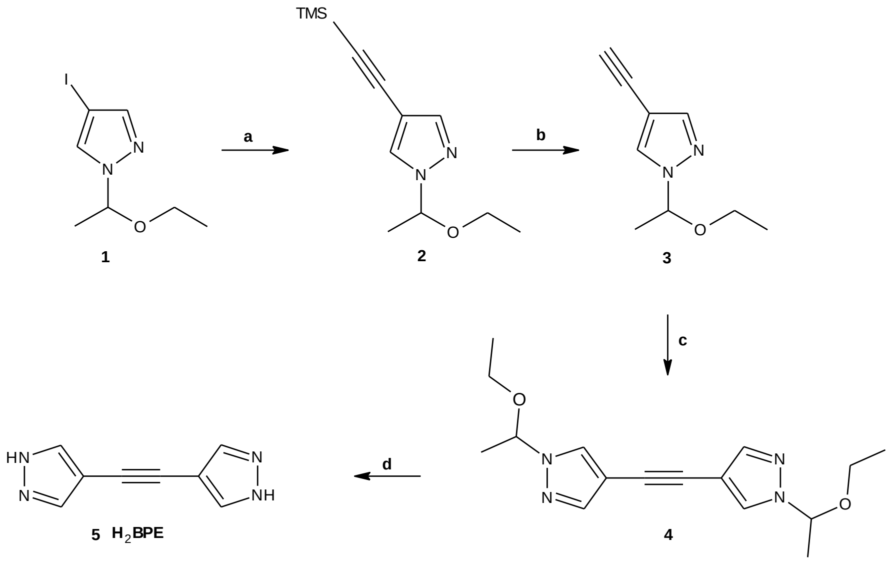

Scheme S1. Synthetic path to prepare the 1,2-di(1*H*-pyrazol-4-yl)ethyne ligand (H_2BPE). a) TMSA, CuI, $(PPh_3)_2PdCl_2$, TEA/THF, toluene, 343 K, Ar; b) K_2CO_3, THF/MeOH/H_2O, 298 K; c) **2**, CuI, $(PPh_3)_2PdCl_2$, 4-Iodo-1-(1-ethoxyethyl)-1*H*-pyrazole,TEA, 343 K, Ar; d) 1,4-dioxane, 6 M $HCl_{(aq)}$, 298 K. TMSA = trimethylsilacetylene; TEA = triethylamine; THF = tetrahydrofurane; MeOH = methanol.

S1.1. Synthesis of 1-(1-ethoxyethyl)-4-iodo-1*H*-pyrazole, 1

4-Iodo-1-(1-ethoxyethyl)-1*H*-pyrazole, **1**, was synthesized according to the procedure described in the literature.[1]

S1.2. Synthesis of 1-(1-ethoxyethyl)-4-((trimethylsilyl)ethynyl)-1*H*-pyrazole, 2

A mixture of THF/TEA (40 mL, 1:1 v/v) was degassed under an argon flow for 20 min. Then **1** (5.0 g, 18.9 mmol) was added, followed by the addition of CuI (420 mg, 2.1 mmol) and $PdCl_2(PPh_3)_2$ (720 mg, 1 mmol). The yellowish solution thus obtained was purged by bubbling argon at room temperature for 5 min. Afterwards, TMSA (3 mL, 21 mmol) was added, and the resulting brown mixture was heated to 333 K and kept at this temperature under stirring. The extent of the reaction was controlled by gas chromatography-mass spectrometry (GC-MS). Additional aliquots of TMSA (100 μL each) were periodically added. After 12 h, GC-MS showed complete consumption of **1**. The dark solution was then cooled down to room temperature and filtered. The brown solid was washed repeatedly with ethyl

acetate (EtOAc). All the liquid fractions thus obtained were combined and dried by rotary evaporation to obtain a brown oil. The latter was dissolved in EtOAc (100 mL) and washed with aqueous ammonium hydroxide (28 wt/wt % NH_3, 2×50 mL) and distilled water (50 mL). The organic layer was dried over Na_2SO_4 and concentrated under reduced pressure. The brown residue was purified by silica-gel gravimetric chromatography (hexane/EtOAc 8:2 v/v, RF = 0.70) to afford **2** as a light brown liquid (4.00 g, 16.9 mmol). Yield = 61%. GC-MS: 236 (M^+) m/z. ^{1}H NMR (400 MHz, DMSO-d_6) δ(ppm): 7.75 (1H, s); 7.61 (1H, s); 5.40 (1H, q, J = 6 Hz); 3.33 (2H, m); 1.65 (3H, d, J = 6 Hz); 1.16 (3H, t, J = 7 Hz); 0.52 (9H, s). ^{13}C NMR (100 MHz, DMSO-d_6) δ(ppm): 0.0, 14.8, 22.1, 64.2, 77.1, 78.4, 87.8, 95.7, 129.6, 141.7.

S1.3. Synthesis of 1-(1-ethoxyethyl)-4-ethynyl-1*H*-pyrazole, 3

Compound **2** (3.04 g, 13 mmol) was dissolved in a mixture of methanol/THF (20 mL, 1:1 v/v). Then, a solution of K_2CO_3 (3.45 g, 26 mmol/40 mL) was added. The mixture was heated to reflux. After 4 h, GC-MS showed complete consumption of **2**. The mixture was then cooled down to room temperature. The organic layer was dried over Na_2SO_4 and concentrated under reduced pressure. The resulting liquid was purified by silica-gel gravimetric chromatography (hexane/EtOAc 8:2 v/v, RF = 0.63) to give **3** as an orange oil. Yield = 55%. GC-MS: 164 (M^+) m/z. ^{1}H NMR (400 MHz, DMSO-d_6) δ(ppm): 7.77 (1H, s); 7.61 (1H, s); 5.50 (1H, q, J = 6 Hz); 3.40 (2H, m); 3.04 (1H, s); 1.63 (3H, d, J = 7 Hz); 1.12 (3H, t, J = 7 Hz). ^{13}C NMR (100 MHz, DMSO-d_6) δ(ppm): 14.7, 22.0, 64.2, 77.0, 78.4, 87.8, 102.0, 129.6, 141.7.

S1.4. Synthesis of 1,2-bis(1-(1-ethoxyethyl)-1*H*-pyrazol-4-yl)ethyne, 4

A mixture of THF/TEA (40 mL, 1:1 v/v) was degassed under an argon flow for 15 min. Then compound **1** (3.24 g, 1.2 mmol), CuI (0.290 g, 1.5 mmol) and $Pd(PPh_3)_4$ (0.274 g, 0.24 mmol) were added. The obtained yellowish solution was purged by bubbling argon at room temperature for 5 min; afterwards, the intermediate **3** (1.77 g, 1.1 mmol) was added and the resulting mixture was heated to 333 K and kept at this temperature under argon and magnetic stirring. The color of the mixture changed from pale yellow to dark brown as the temperature was raised. After 6 h, the GC-MS analysis showed the complete consumption of the reagents. The mixture was cooled down to room temperature and then filtered on a Hirsch funnel; the filtrate was dried by rotary evaporation giving an orange/brown liquid. The latter was dissolved in EtOAc and washed with aqueous ammonium hydroxide (28 wt/wt % NH_3, 2×25 mL) and distilled water (25 mL). The organic phase was dried over Na_2SO_4 and concentrated under vacuum to afford a brown solid. The latter was purified by silica-gel gravimetric chromatography with a tertiary eluent (hexane/AcOEt/dichloromethane 7:2:1 v/v/v) to afford the title compound **4** as a white solid. GC-MS: 302.37 (M^+) m/z. ^{1}H NMR (400 MHz, DMSO-d_6) δ(ppm): 8.22 (2H, s); 7.69 (2H, s); 5.55 (2H, q, J = 8 Hz); 3.32 (2H, m); 1.59 (6H, d, J = 8 Hz); 1.04 (6H, t, J = 8 Hz). ^{13}C NMR (100 ppm, DMSO-d_6) δ(ppm): 141.4, 131.4, 103.1, 87.0, 81.9, 63.6, 21.6, 15.1. IR(ATR) ν

(cm^{-1}): 3129 (vw); 3109 (w); 3086 (vw); 2981 (m); 2925 (w); 2880 (w); 1581 (w); 1439 (m); 1373 (s); 1335 (m); 1260 (m); 1174 (s); 1120 (vs); 1092 (s); 1023 (m); 980 (m); 857 (m); 754 (m); 680 (vw); 660 (w); 636 (m). Elemental analysis (%) for $C_8H_{22}N_4O_2$ (FW = 302.37 a.m.u.), calc.: C 63.55, H 7.33, N 18.5; found: C 62.83, H 7.51, N 17.93.

S1.5. Synthesis of 1,2-bis(1*H*-pyrazol-4-yl)ethyne, H$_2$BPE

Compound **4** (1.70 g, 0.85 mol) was dissolved in 1,4-dioxane (7 mL). Five drops of concentrated $HCl_{(aq)}$ (37% m/m) were then added. A white solid precipitated very quickly. The reaction mixture was heated to 313 K and kept at this temperature under magnetic stirring for 4 h. Then the precipitate was filtered, washed with 1,4-dioxane (5 mL) and methanol (20 mL) and dried under vacuum overnight to afford the title compound as a white solid. ^{1}H NMR (400 MHz, DMSO-d_6) δ(ppm): 7.86 (s, 4H); 5.96 (s br 2H, N-H). ^{13}C NMR (100 MHz, DMSO-d_6) δ(ppm): 135.9, 101.5, 81.6. IR (ATR) ν(cm^{-1}): 3116 (m); 3030 (w); 2958 (m); 2854 (m); 2500 (vs, b); 1558 (s); 1471, (m); 1373 (s); 1292 (w); 1225 (m); 1137 (m); 1088 (m); 1005 (w); 896 (s); 719 (m); 625 (s). Elemental analysis (%) for $C_8H_6N_4$ (FW = 302.37 a.m.u.), calc.: C = 60.76, H = 3.82, N = 35.42; found: C = 59.93, H = 3.88, N = 34.24.

S2. Infrared spectroscopy

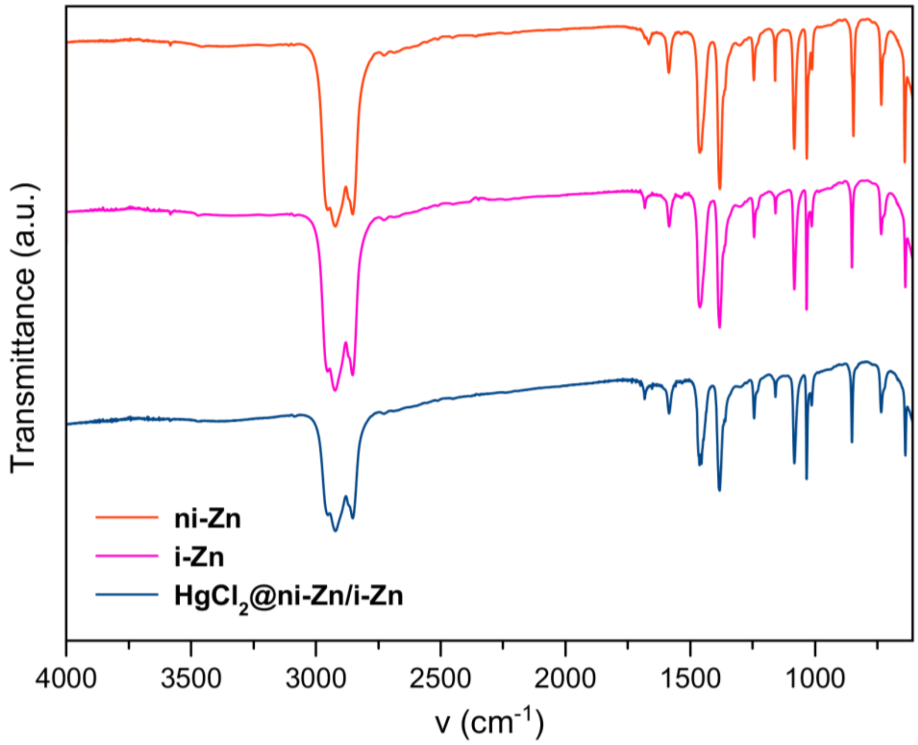

Figure S1. Infrared spectra of ni-Zn, i-Zn and a sample of the $HgCl_2$@ni-Zn/i-Zn mixture (magenta, red and blue traces, respectively) acquired in Fourier transform mode.

S3. Powder X-ray diffraction structural characterization

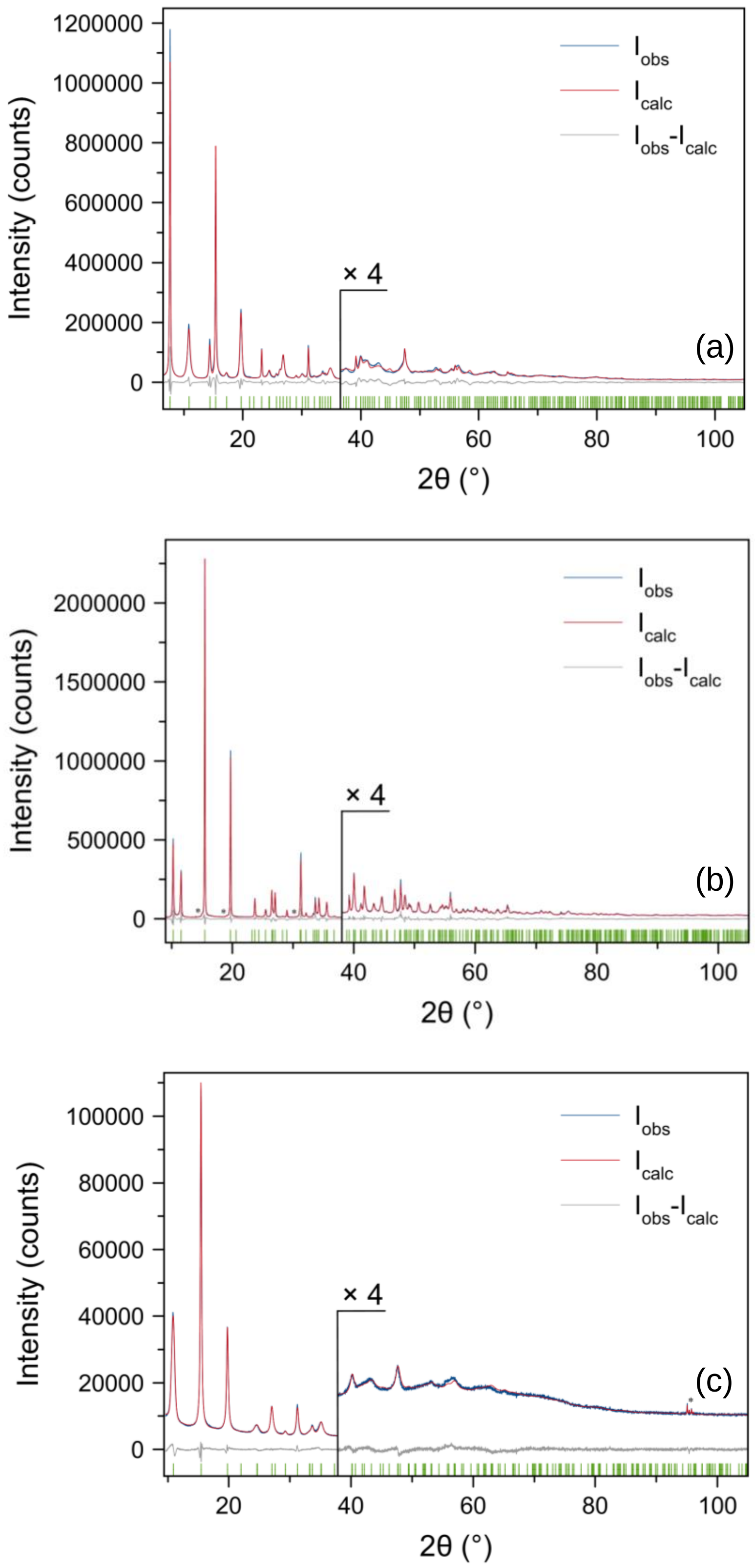

Figure S2. Graphical result of the final Rietveld refinements carried out on the PXRD data of (a) ni-Zn·S, (b) i-Zn and (c) t-i-Zn in terms of experimental, calculated and difference traces (blue, red and grey, respectively). The green markers at the bottom indicate the positions of the Bragg reflections. The asterisks indicate peaks belonging to (b) impurities or (c) the silicon free-background sample-holder.

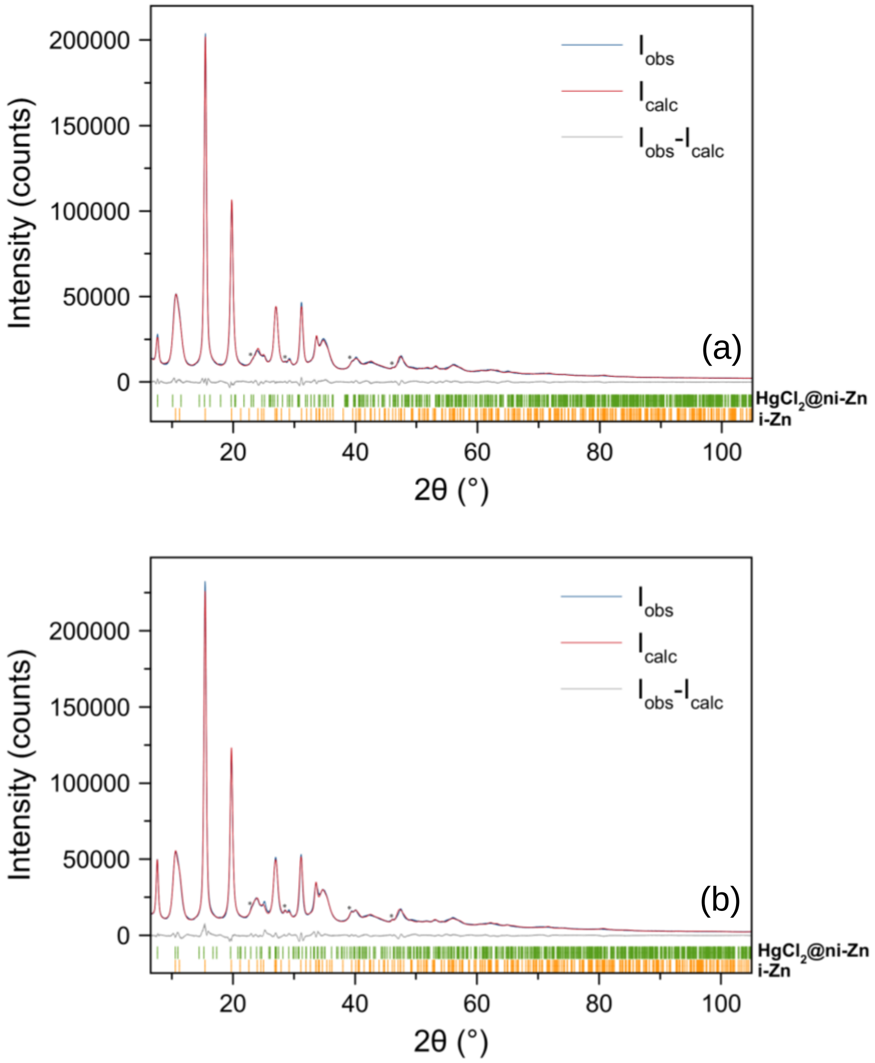

Figure S3. Graphical result of the final Rietveld refinements carried out on the PXRD data of a $HgCl_2$@ni-Zn/i-Zn sample obtained upon suspending i-Zn in $HgCl_{2(aq)}$ (a) 300 ppm for 1 h and (b) 500 ppm for 2 h (see the Experimental Section in the main text). Experimental, calculated and difference traces (blue, red and grey, respectively). The orange and green markers at the bottom indicate the positions of the Bragg reflections for i-Zn and $HgCl_2$@ni-Zn, respectively. The asterisks indicate peaks belonging to impurities.

Table S1. Details of the $HgCl_2$ clathration experiments carried out as described in the Experimental Section.

$[HgCl_2]$ (ppm)	$[HgCl_2]$ (mmol/L)	n_i(i-Zn) (mmol)	$n_i(HgCl_2)$ (mmol)	n_i(i-Zn)/$n_i(HgCl_2)$
5000	18.42	9.0×10^{-2}	3.7×10^{-2}	2
2000	7.37	9.0×10^{-2}	1.5×10^{-2}	6
1000	3.68	9.0×10^{-2}	7.4×10^{-3}	12
500	1.84	9.0×10^{-2}	3.7×10^{-3}	25
400	1.47	9.0×10^{-2}	2.9×10^{-3}	31
300	1.11	9.0×10^{-2}	2.2×10^{-3}	41
200	0.74	9.0×10^{-2}	1.5×10^{-3}	61
100	0.37	9.0×10^{-2}	7.4×10^{-4}	123
75	0.28	9.0×10^{-2}	5.5×10^{-4}	163
50	0.18	9.0×10^{-2}	3.7×10^{-4}	245
10	0.04	9.0×10^{-2}	7.4×10^{-5}	1226

Table S2. Details of the Rietveld refinements carried out on the $HgCl_2$@ni-Zn/i-Zn samples as described in the Experimental Section.

Sample	Data	Param.	R_p	R_{wp}	R_{Bragg}	% m/m Hg@ni-Zn	mol($HgCl_2$)/f.u.	x, y, z Hg
5000 ppm, 1 h	4951	98	0.026	0.033	0.003	81.6	0.29	0.6393(1), 0.89645(7), 0.6642(4)
2000 ppm, 1 h	4951	80	0.028	0.037	0.003	47.4	0.32	0.6439(2), 0.9011(1), 0.6693(4)
1000 ppm, 1 h	4951	78	0.031	0.040	0.010	33.6	0.33	0.6180(2), 0.8724(2), 0.6764(6)
500 ppm, 4 h	4951	67	0.026	0.034	0.003	20.0	0.27	0.1255(3), 0.3920(2), 0.603(1)
500 ppm, 2 h	4951	67	0.031	0.041	0.004	19.6	0.29	0.1232(4), 0.3899(2), 0.6110(8)
500 ppm, 1 h	4951	62	0.025	0.033	0.003	19.4	0.28	0.1284(3), 0.3925(2), 0.616(2)
500 ppm, 30 min	4951	63	0.032	0.042	0.004	19.6	0.27	0.1206(3), 0.3807(3), 0.649(1)
500 ppm, 10 min	4951	67	0.026	0.033	0.002	19.4	0.26	0.0752(2), 0.3536(4), 0.552(1)
400 ppm, 1 h	4951	62	0.021	0.028	0.002	15.4	0.25	0.1270(4), 0.3951(2), 0.600(3)
300 ppm, 1 h	4951	64	0.026	0.033	0.005	11.9	0.26	0.1059(4), 0.3791(3), 0.534(2)
200 ppm, 1 h	4951	55	0.026	0.034	0.007	8.4	0.25	0.1475(5), 0.4122(3), 0.635(2)
100 ppm, 1 h	4951	47	0.028	0.037	0.012	3.9	0.24	0.1264(5), 0.4055(6), 0.616(3)

Table S3. Details of the alkaline and earth-alkaline metal chloride solutions prepared for this work.

[metal chloride] (mmol/L)	[$HgCl_2$] (ppm)	[NaCl] (ppm)	[KCl] (ppm)	[$MgCl_2 \cdot 6H_2O$] (ppm)	[$CaCl_2 \cdot 2H_2O$] (ppm)
1.8	500	108	137	374	271

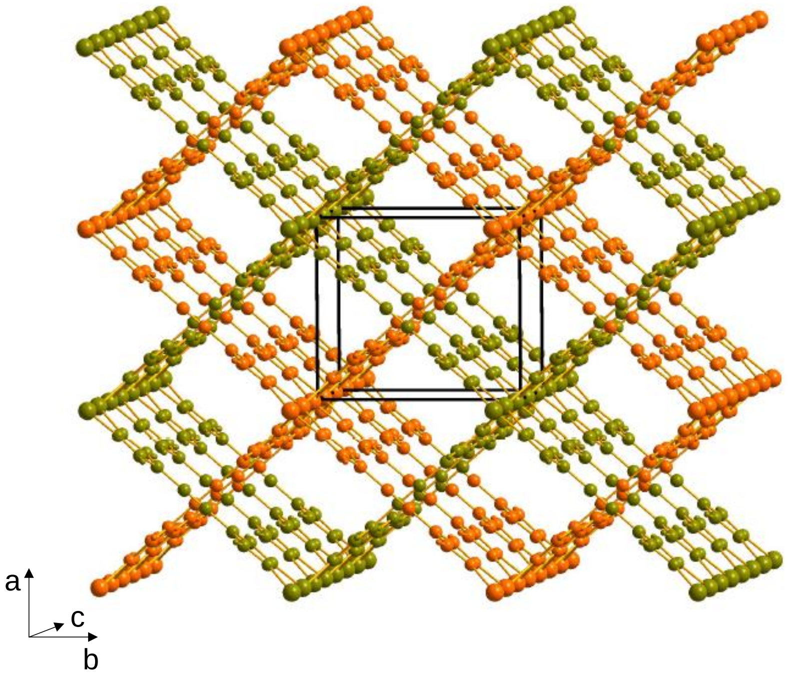

Figure S4. Portion of the two interpenetrated networks characterizing the crystal structure of i-Zn (represented with different colours for the sake of clarity) viewed in perspective along the [001] crystallographic direction.

S4. Thermal behaviour

As shown by the TGA and DTG traces (Figure S5), the ligand is stable up to ~465 K. Decomposition occurs in a rather turbulent way with four consecutive weight losses yielding a black carbonaceous residue at temperatures above 800 K. Due to this observation, no STA was carried out on the two MOFs.

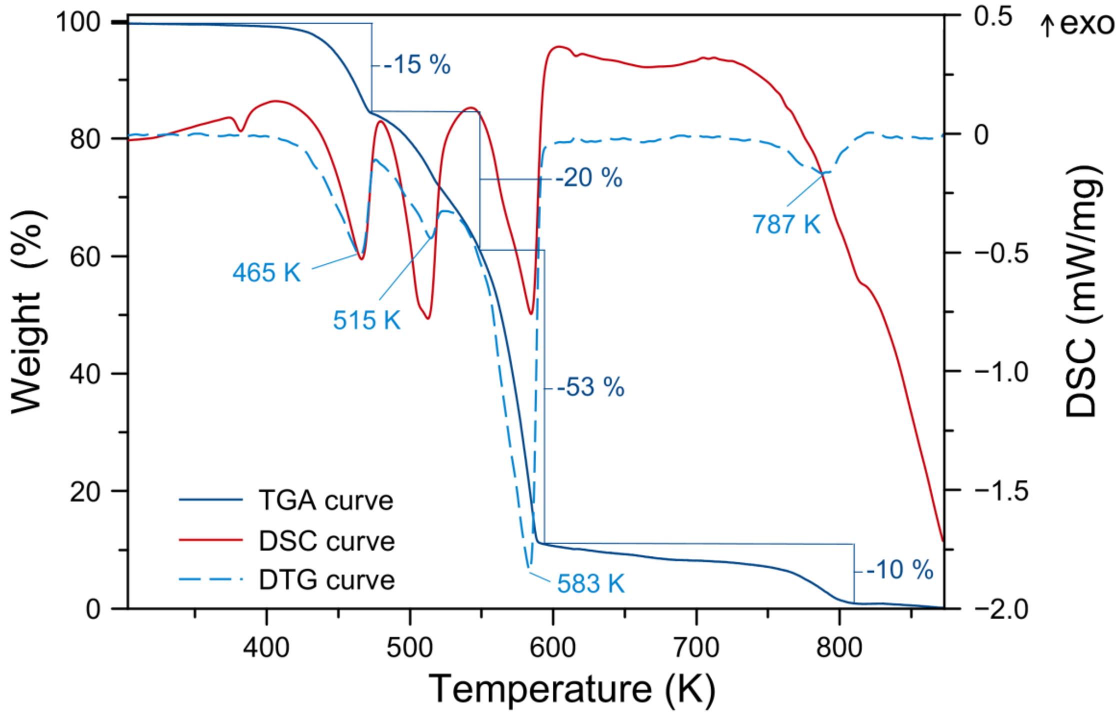

Figure S5. Simultaneous thermal analysis of the H_2BPE ligand performed under a N_2 flow at a 10 K/min rate.

The thermal behaviour of ni-Zn and i-Zn was studied by *in situ* variable-temperature PXRD (VT-PXRD) experiments carried out in air. The latter highlighted that ni-Zn and i-Zn are stable up to 723 K and 763 K, respectively (Figures S6a and S6b, respectively). In the case of ni-Zn, no phase changes are observed in the investigated temperature range. The decrease, by 1.3%, of the unit cell volume in the range 298-703 K (volumetric thermal expansion coefficient α_v = -33 MK^{-1}) (Figure S7a) is indicative of a certain framework rigidity. On the other hand, for i-Zn, starting from 303 K the *a*/*b* ratio progressively increases from the value of ~0.9 upon increasing the temperature. At ~483 K the *a*-axis equals the *b*-axis, witnessing the crystallographic symmetry increase from orthorhombic to tetragonal. The tetragonal polymorph (t-i-Zn) is stable up to ~763 K and is more flexible than the orthorhombic polymorph (ΔV = 0.8% and -2.6%, α_v = 48 and -110 MK^{-1} in the 303-463 and 483-723 K ranges for the orthorhombic and tetragonal polymorphs, respectively) (Figure S7b).[2] t-i-Zn can be recovered also upon heating *ex situ* in an oven (573 K, 30 min) a sample of i-Zn and is stable under ambient conditions. Based on the structural characterization, t-i-Zn crystallizes in the space group $P4_2/mcm$ and possesses

the same structural motif of the orthorhombic counterpart, with square rather than rhombic channels (see Figure S1c for the final refinement of the crystal structure).

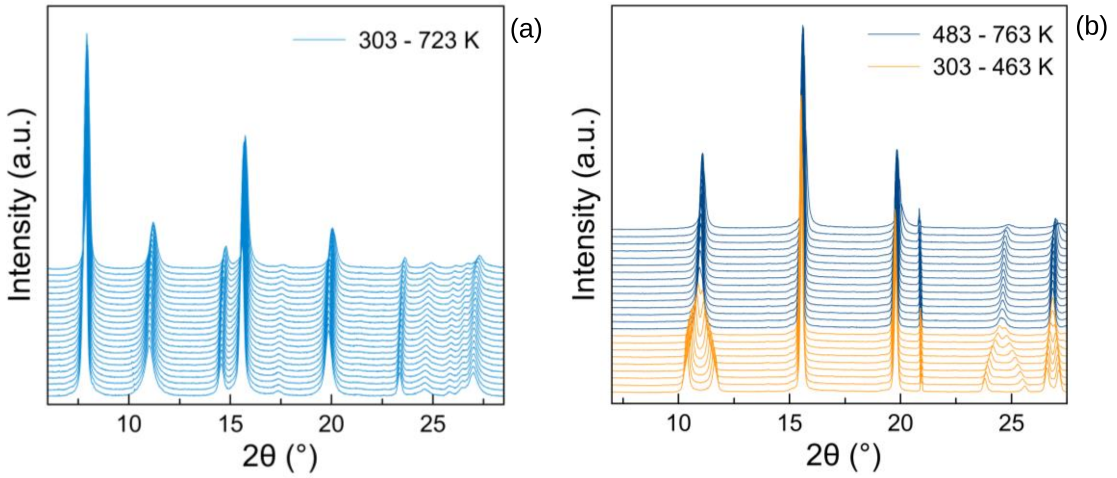

Figure S6. Powder X-ray diffraction patterns acquired, as a function of the temperature and with steps of 20 K, on (a) ni-Zn·S, in the temperature range 303-723 K (from bottom to top); (b) i-Zn, in the temperature range 303-463 K (from bottom to top, orange traces), and t-i-Zn, in the temperature range 483-763 K (from bottom to top, blue traces).

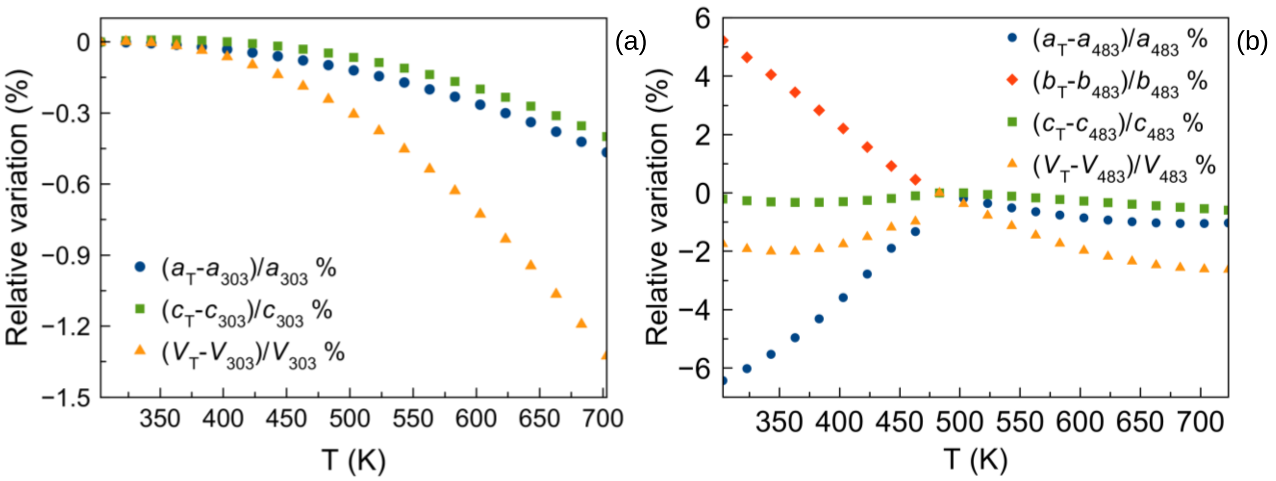

Figure S7. Percentage relative variation of the unit cell parameters in (a) ni-Zn·S and (b) i-Zn as a function of the temperature, as retrieved by a parametric whole powder pattern refinement on the VT-PXRD patterns shown in Figure S6. Note that the values reported in (b) are normalized with respect to the unit cell parameters of the tetragonal phase detected at 483 K.

S5. Textural properties

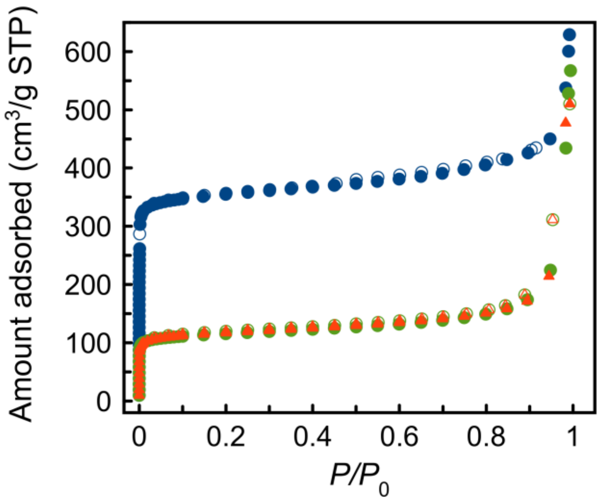

Figure S8. N_2 adsorption isotherms acquired at 77 K on ni-Zn (blue circles), i-Zn (red triangles) and ni-Zn after suspension in water (green circles); empty symbols depict the desorption branch.

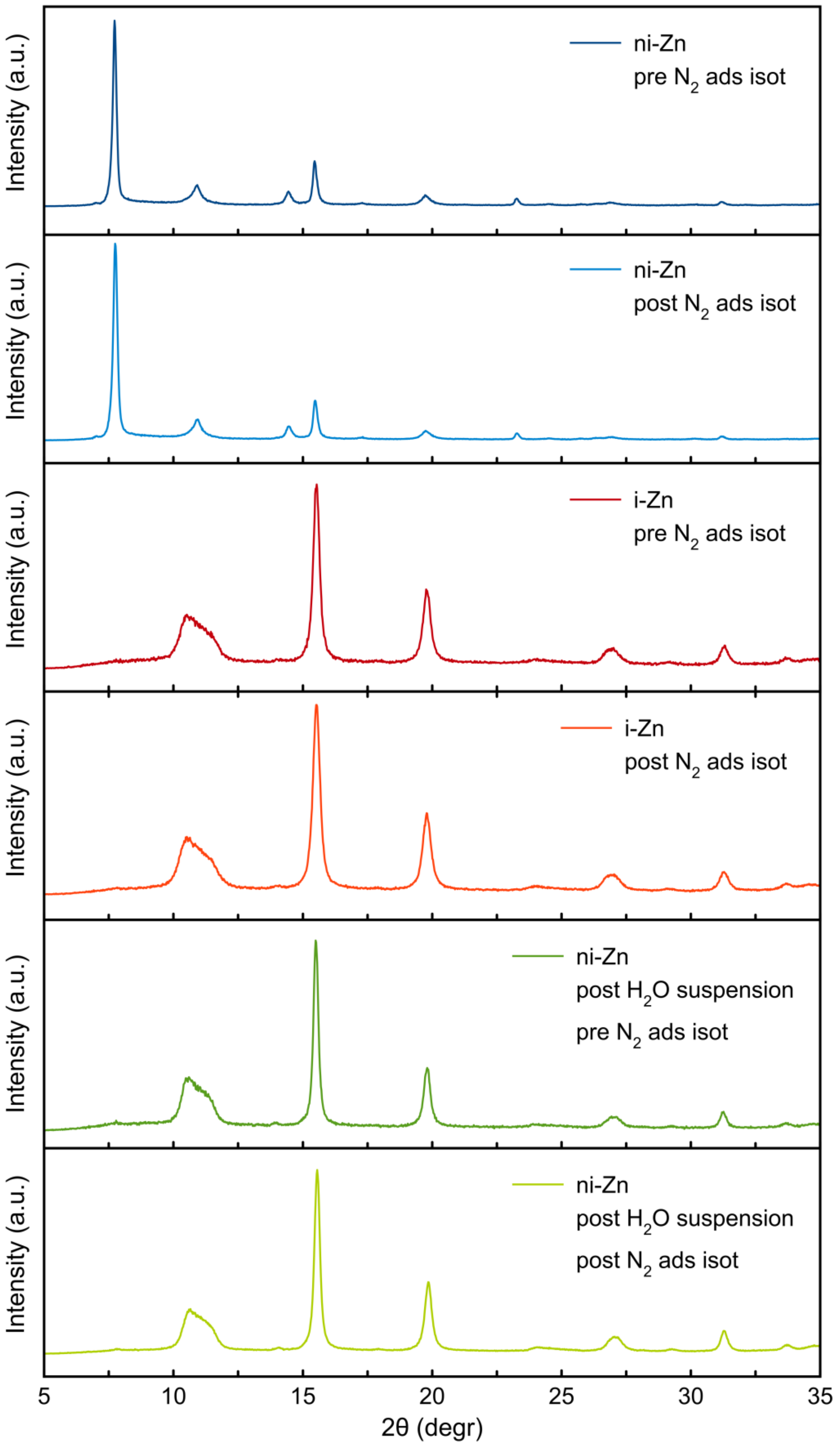

Figure S9. Comparison of the PXRD patterns of ni-Zn, i-Zn and ni-Zn suspended in water before and after the acquisition of the N_2 adsorption isotherms at 77 K.

S6. Water stability

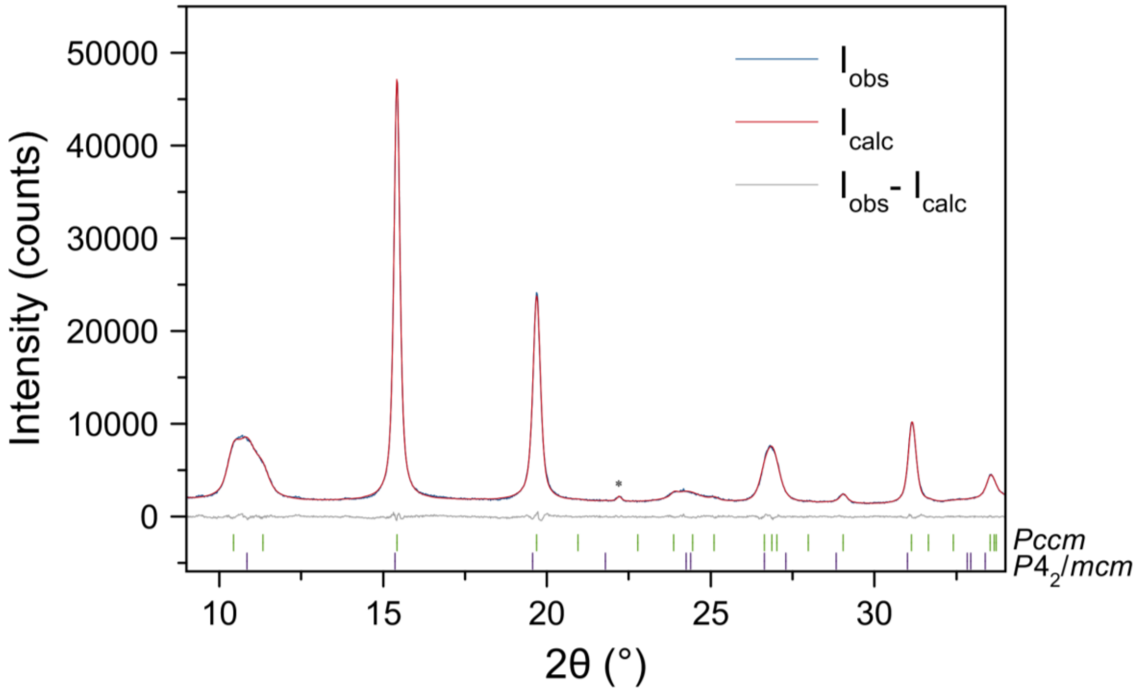

Figure S10. Graphical result of the whole powder pattern refinement carried out with the Le Bail approach on the PXRD pattern of ni-Zn·S after 24 h of suspension in water. Experimental, calculated and difference profiles: blue, red and grey, respectively. Peak maxima positions of the interpenetrated orthorhombic *Pccm* phase, cyan ticks; peak maxima positions of the interpenetrated tetragonal $P4_2/mcm$ phase: orange ticks. R_p = 0.020; R_{wp} = 0.027. The asterisk indicates a peak belonging to an impurity.

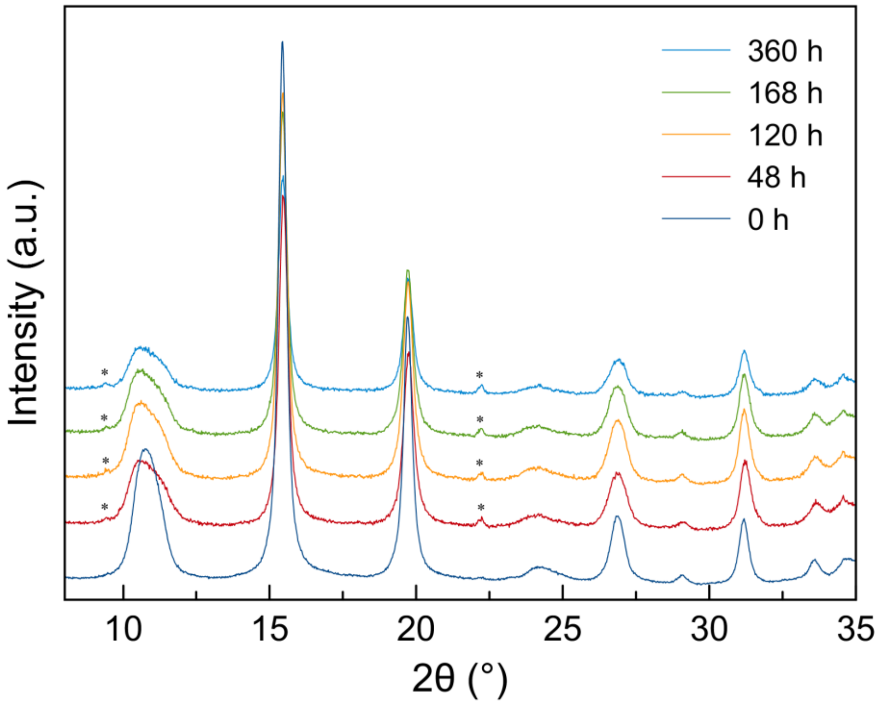

Figure S11. Powder X-ray diffraction patterns acquired, at different time lapses, on i-Zn during the water suspension experiment. The asterisks indicate peaks belonging to an impurity.

S7. Theoretical calculations

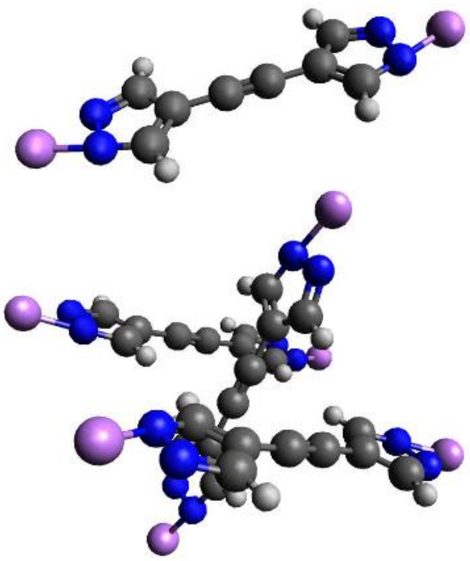

Figure S12. Optimized geometries for the models of the non-interpenetrated (top) and interpenetrated (bottom) frameworks employed to investigate possible interactions modes for $HgCl_2$. Atoms colour code: carbon, grey; hydrogen, light grey; lithium, violet; nitrogen, blue.

S8. Electronic-state transition spectroscopy

The spectral line-shapes of the ligand (blue) and of the i-Zn and ni-Zn·S MOFs (magenta and red, respectively) are plotted in Figure S13. The three compounds exhibit intense absorption in the UVB portion of the spectrum. Namely, a main absorption band is present with barycentre around 250 nm. Two well defined peaks are resolvable within the band, with maxima at ~230 and ~265 nm. Both peaks exhibit a residual substructure. The first peak can be attributed to the absorption of the pyrazole chromophore (the absorption peak of the isolated ring falls at 210 nm[3]), while the second one pertains to the π-π* transition involving the central carbon-carbon triple bond (the isolated chromophore exhibits an absorption maximum at 230 nm[4]). According to these attributions, the chromophores energy levels are perturbed by both incorporation within the H_2BPE molecule and insertion in the crystal structure of the two MOFs: the measurable effect is a ~20-30 nm red shift of all the absorption bands, indicating a sizeable degree of intra- and inter-molecular charge conjugation. Coordination to the metal ion does not bring about qualitative changes in the ligand absorption photophysics. Particularly, the onset of bands ascribable to ligand-to-metal charge-transfer dynamics, which are frequently enabled by incorporation of organic molecules in coordination compounds, is not observable in the present instance, possibly due to a non-negligible baseline absorption in the UVA portion of the spectrum, *i.e.* in the energy range in which these transitions are usually elicited. However, the spectrum of ni-Zn is sharper, indicating an overall reduction in the vibrational degrees of freedom upon metal ion coordination. Conversely, interpenetration partially restores the ligand spectrum broadness for i-Zn, suggesting the instauration of π-π stacking interactions between the alkyl moieties of neighbouring ligands, where these orbitals are optimally permeating to one another, and the consequent enablement of synergistic vibrational modes.

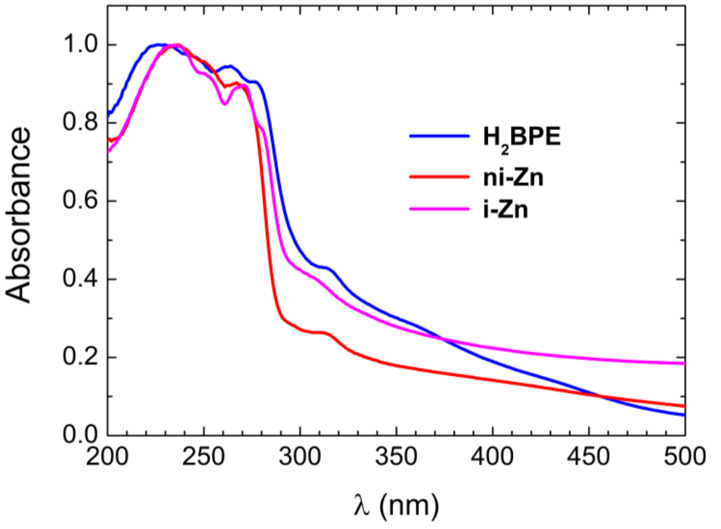

Figure S13. Peak-normalized UV-Vis absorption spectral line-shapes of the H_2BPE ligand (blue line) and of the ni-Zn·S (red line) and i-Zn (magenta line) MOFs.

Sizeable fluorescence is excited for the ligand as well as the two MOFs by irradiation in correspondence of both the pyrazole and the alkyl absorption bands. Upon excitation at 230 nm, a fainter fluorescence is observed, as denounced by the noisier profile of the spectral line-shape (Figure S14a). The spectra exhibit two peaks at 400 nm and 470 nm, the latter being relatively more intense for the i-Zn MOF. When the three compounds are excited at 265 nm, the violet emission disappears and the blue one becomes roughly twice more intense (Figure S14b). Besides a slight red shift, more evident in the case of ni-Zn, coordination to Zn^{II} does not induce significant changes in the fluorescence spectral line-shape. The i-Zn emission is more intense than that of both ni-Zn and H_2BPE.

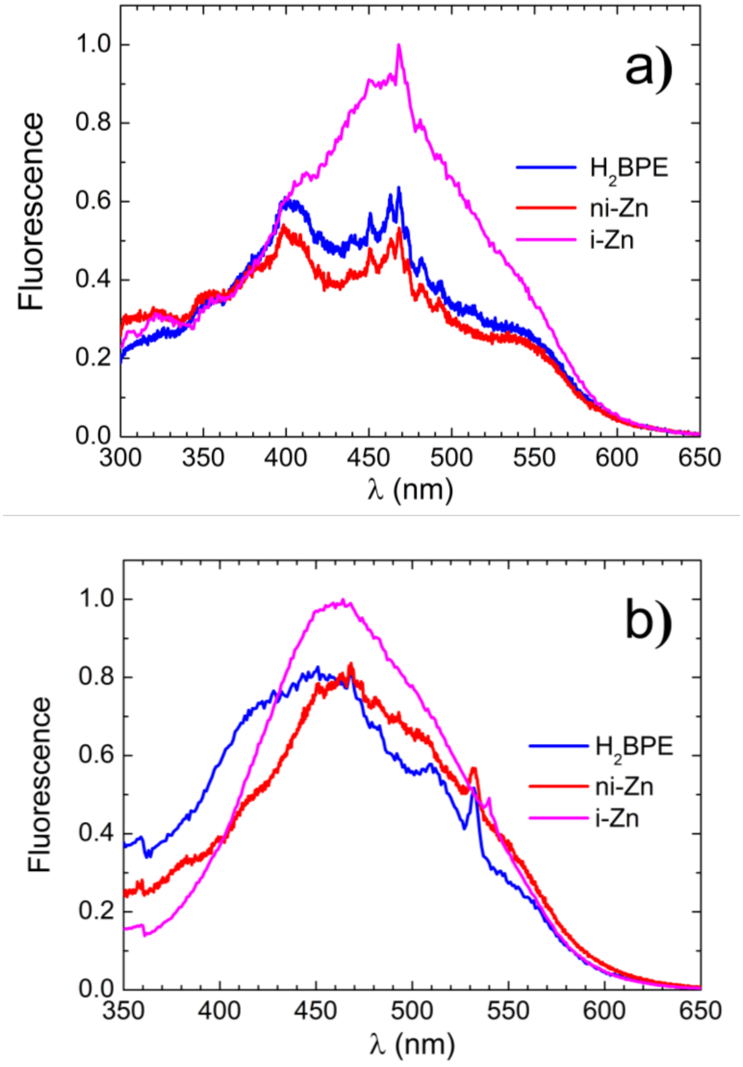

Figure S14. Fluorescence emission spectra of the H_2BPE ligand (blue line) and of the ni-Zn (red line) and i-Zn (magenta line) MOFs upon excitation at (a) 230 nm and (b) 265 nm. The spectra were normalized to the i-Zn emission peak.

With the aim of investigating the fine structure of the absorption band of the two MOFs, we recorded their fluorescence excitation spectra, acquiring the fluorescence emitted at the fixed observation wavelength of 470 nm as a function of the excitation wavelength in the region 250-400

nm. Unexpectedly, as shown in Figure S15a, excitation of ni-Zn in correspondence of the red tail of the absorption spectrum results in a notable increase in fluorescence emission, while in the case of i-Zn a much fainter fluorescence is attained upon excitation above 290 nm. We interpreted this fact as due to the elicitation of charge-transfer transitions upon irradiation in this portion of the spectrum. Indeed, the well-resolved excitation band peaked at ≈365 nm appearing in both spectra constitutes a clear spectroscopic benchmark of at least one of these transitions, the presence of which, although not evidenced by the absorption spectra of Figure S13, is confirmed by *in silico* calculations (see the main text).

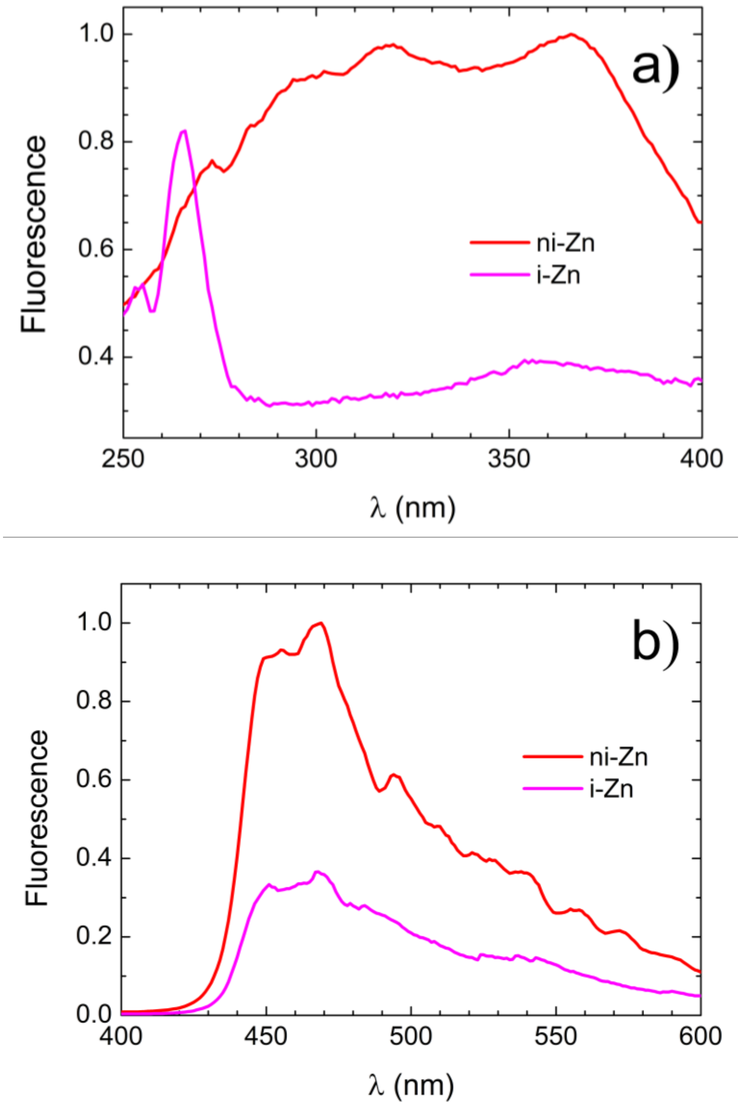

Figure S15. (a) Fluorescence excitation spectra of the ni-Zn (red line) and i-Zn (magenta line) MOFs acquired at λ_{obs} = 470 nm. (b) Fluorescence emission spectra of the ni-Zn (red line) and i-Zn (magenta line) MOFs acquired with λ_{exc} = 365 nm. The spectra were normalized to the ni-Zn excitation and emission peaks in Figures S14a and S14b, respectively.

The ni-Zn spectrum also exhibits additional peaks at ≈300 nm and ≈320 nm, suggesting a rather complex metal-to-ligand charge exchange pattern at least for this compound. Although the excitation spectra of the two MOFs maximally differ in correspondence of the 320 nm peak, we deemed that, in view of sensing applications, exploiting an excitation at the edge of the visible band would result in safety as well as technical advantages. Indeed, prolonged exposure to radiation at 320 nm might be harmful for the health. Moreover, this wavelength is notably absorbed by most glasses and plastics. Thus, we undertook a full characterization of the effects of $HgCl_2$ on the i-Zn/ni-Zn fluorescence emission exciting at 365 nm, which conversely can be provided by standard, low cost UVB lamps of the kind, *e.g.*, of those used for the photopolymerization of resins. Preliminarily to this task, the fluorescence emission spectra obtained upon excitation at this wavelength for as-synthesized i-Zn and ni-Zn were acquired (Figure S15b). Interestingly, the spectral line-shape is notably structured. In particular, it exhibits an additional peak at ≈450 nm and a wealth of vibrational modulations in the long-wavelength tail. These features suggest that completely different excited-state dynamics are in act with respect to that observed upon direct excitation of the alkyl π-π* transition resonance band at 265 nm and confirm the complexity of the charge-transfer dynamics. The emission intensity is two-to-three-fold reduced for i-Zn with respect to ni-Zn, in agreement with the indications offered by the corresponding excitation spectra. Once again, the observed fluorescence quenching might correlate with the π-π stacking interactions between the alkyl moieties of neighbouring spacers in i-Zn. Such interactions are indeed known to provide efficient non-radiative decay pathways. Time-correlated single-photon counting (TCSPC) measurements support the picture of faster non-radiative decay being the main cause of the dimmer fluorescence of i-Zn. Both ni-Zn and i-Zn decay through double-exponential patterns (see Table S4). However, the relative amplitude of the faster decay component is higher for i-Zn than for ni-Zn. Moreover, both decay times are significantly longer for ni-Zn. The result is a longer-lived fluorescence emission in the former case, as evidenced by the decay patterns shown in Figure S16 (red and magenta plots for ni-Zn and i-Zn, respectively).

Table S4. Fitting parameters retrieved from a double-exponential fit of the experimental fluorescence decay patterns of ni-Zn, i-Zn and the $HgCl_2$@ni-Zn/i-Zn system obtained from 500 ppm $HgCl_{2(aq)}$ for 2 h (see the main text for details).

System	f_1	τ_1 (ps)	f_2	τ_2 (ps)
ni-Zn·S	0.433 ± 0.004	644 ± 17	0.567 ± 0.004	3880 ± 92
i-Zn	0.680 ± 0.007	558 ± 3	0.320 ± 0.007	2379 ± 15
$HgCl_2$@ni-Zn/i-Zn, 500 ppm 2 h	0.672 ± 0.003	603 ± 5	0.328± 0.008	2675± 23
$HgCl_2$@ni-Zn/i-Zn, 500 ppm 2 h†	0.662 ± 0.005	647 ± 5	0.260 ± 0.005	2370 ± 7

† Constrained three-exponential fit with τ_3 fixed at 3880 ps. The value attained for f_3, which was left free to vary, was 0.078±0.005.

An exemplary sample recovered after suspension in 500 ppm $HgCl_{2(aq)}$, namely that kept in solution for 2 h (cyan plot), was further investigated by means of time-correlated single-photon counting. Such analysis provides additional fingerprints of the $HgCl_2$-mediated i-Zn-to-ni-Zn conversion. The obtained fluorescence decay pattern is compared to those of as-synthesized i-Zn and ni-Zn in Figure S16. Qualitatively, the fluorescence lifetime is intermediate between that of the two pristine materials. A two-exponential fit permitted to retrieve the fitting parameters collected in Table S4. Interestingly, by attempting a constrained three-exponential fit with the longest-lived component fixed at the value measured for the slowest component resolved in the $HgCl_2$@ni-Zn decay, the intermediate component spontaneously converged, within the experimental errors, to a value equal to that measured for the longest-lived component of i-Zn. This occurrence suggests that the exemplary sample is indeed a mixture of i-Zn and $HgCl_2$@ni-Zn. The mass fraction of $HgCl_2$@ni-Zn can be estimated as the ratio between the relative amplitude of the slowest decay component and the sum of the relative amplitudes of the slowest and intermediate components, and results f_{Fluo}($HgCl_2$@ni-Zn) = 0.23, in good agreement with the fraction determined by PXRD [f_{PXRD}($HgCl_2$@ni-Zn) = 0.20; Table S2]. The short-lived component assumes a value in between those measured for the two as-synthesized MOFs (Table S4).

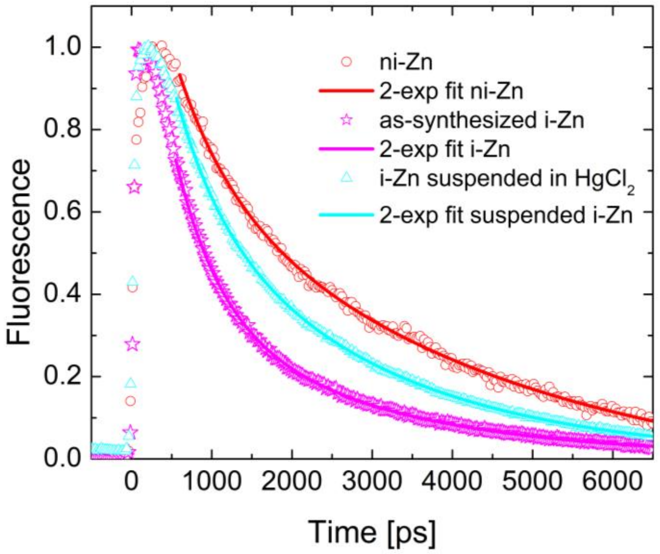

Figure S16. Fluorescence decay patterns of as-synthesized ni-Zn·S (red circles) and i-Zn (magenta crosses), and of the $HgCl_2$@ni-Zn/i-Zn system obtained suspending i-Zn in 500 ppm $HgCl_{2(aq)}$ for 2 h (cyan triangles, see the main text for details). The continuous lines are the best fitting curves to a double-exponential decay model.

S9. $HgCl_2$ adsorption

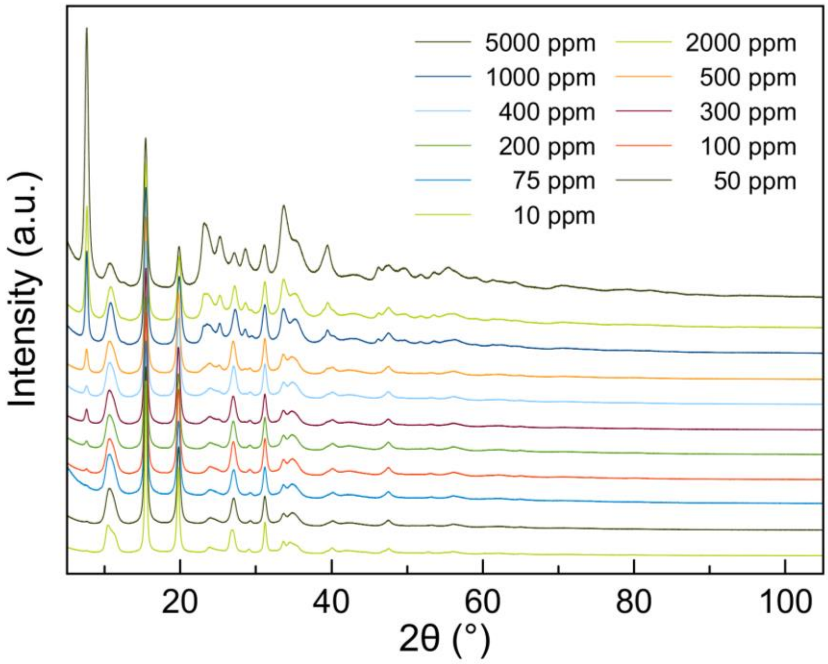

Figure S17. Powder X-ray diffraction patterns of the $HgCl_2$@ni-Zn/i-Zn samples recovered from 1 h suspension of i-Zn in $HgCl_2$ aqueous solutions of concentration in the range 10-5000 ppm.

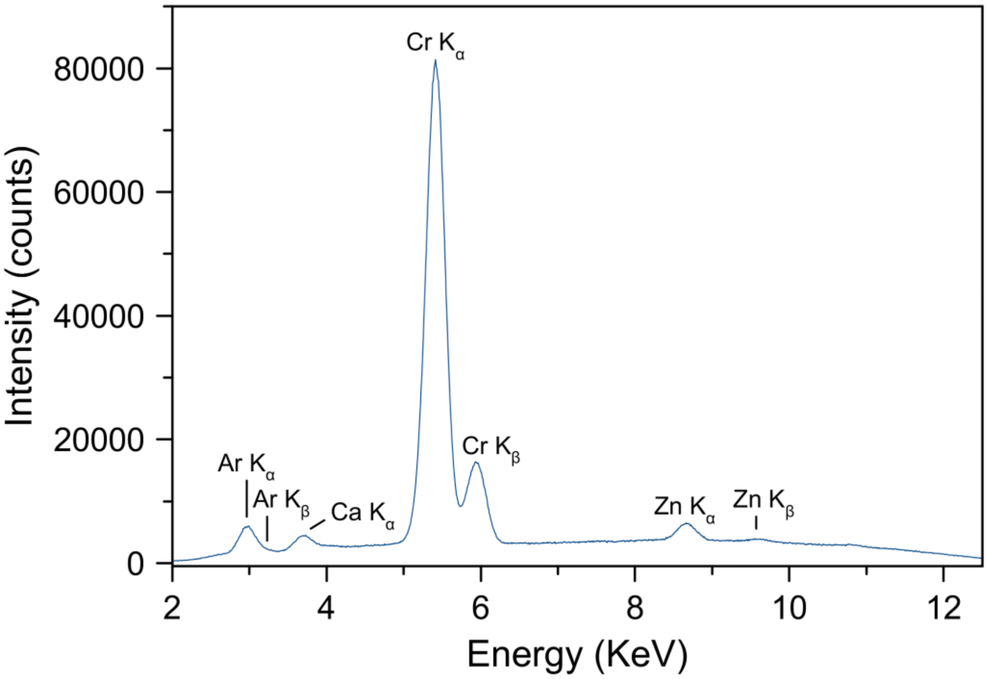

Figure S18. X-ray fluorescence spectrum acquired on the mother liquors recovered after 1 h suspension of i-Zn in $HgCl_{2(aq)}$ 500 ppm. The characteristic lines of argon derive from the fact that the measurement was carried out in air, the characteristic lines of chromium derive from the anode of the X-ray source, while the characteristic line of calcium derives from an impurity present in the polymeric film constituting the base of the sample holder.

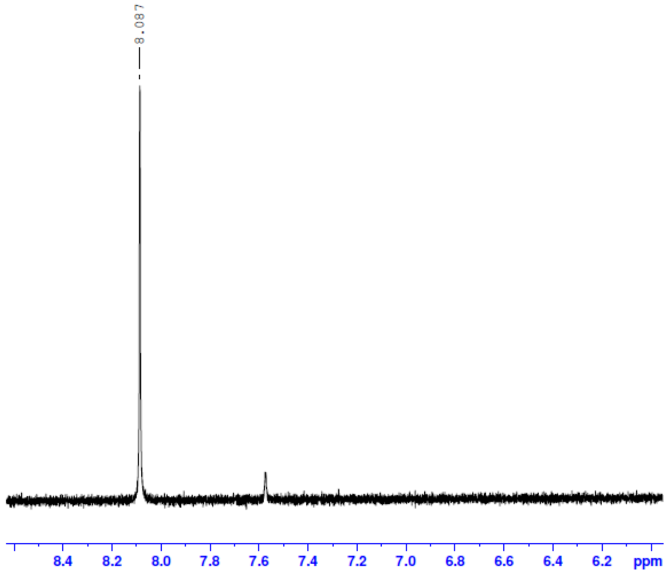

Figure S19. ^{1}H NMR spectrum in D_2O of the mother liquors recovered after 1 h suspension of i-Zn in $HgCl_{2(aq)}$ 500 ppm. The signal at 8.09 ppm belongs to the hydrogen atoms of the heteroaromatic ring, witnessing the presence of the H_2BPE ligand in the mother liquors.

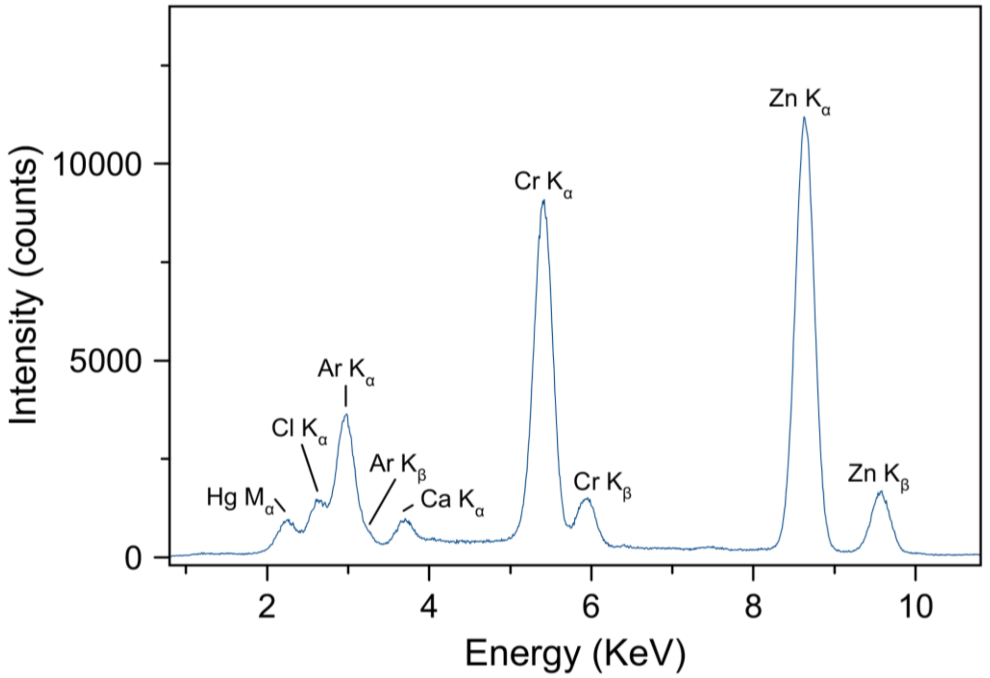

Figure S20. X-ray fluorescence spectrum acquired on the $HgCl_2$@ni-Zn/i-Zn sample obtained upon 1 h suspension of i-Zn in $HgCl_{2(aq)}$ 500 ppm. The characteristic lines of argon derive from the fact that the measurement was carried out in air, the characteristic lines of chromium derive from the anode of the X-ray source, while the characteristic line of calcium derives from an impurity present in the polymeric film constituting the base of the sample holder.

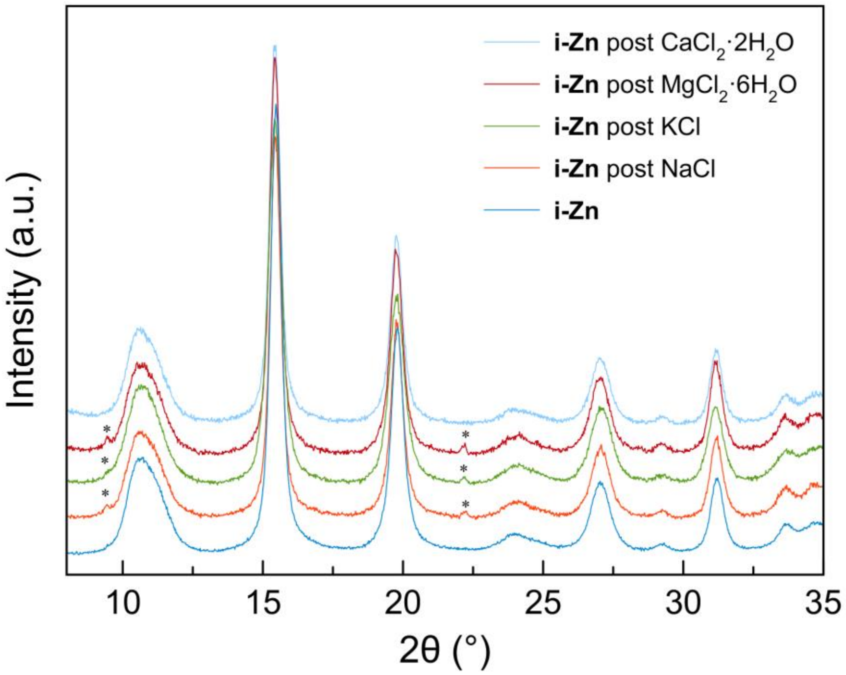

Figure S21. Powder X-ray diffraction patterns of the samples recovered after i-Zn suspension in 1.8×10^{-3} M aqueous solutions (2 mL) of NaCl, KCl, $MgCl_2\cdot6H_2O$ and $CaCl_2\cdot2H_2O$ for 1 h.

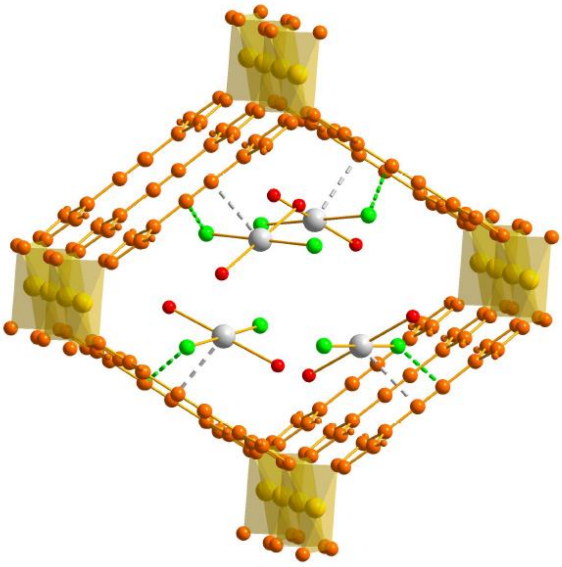

Figure S22. Position of the $HgCl_2$ molecules in the 1-D channels of $HgCl_2$@ni-Zn viewed in perspective along the [001] crystallographic direction. Element colour code: carbon, hydrogen, nitrogen, orange; chlorine, light green; mercury, grey; zinc, gold.

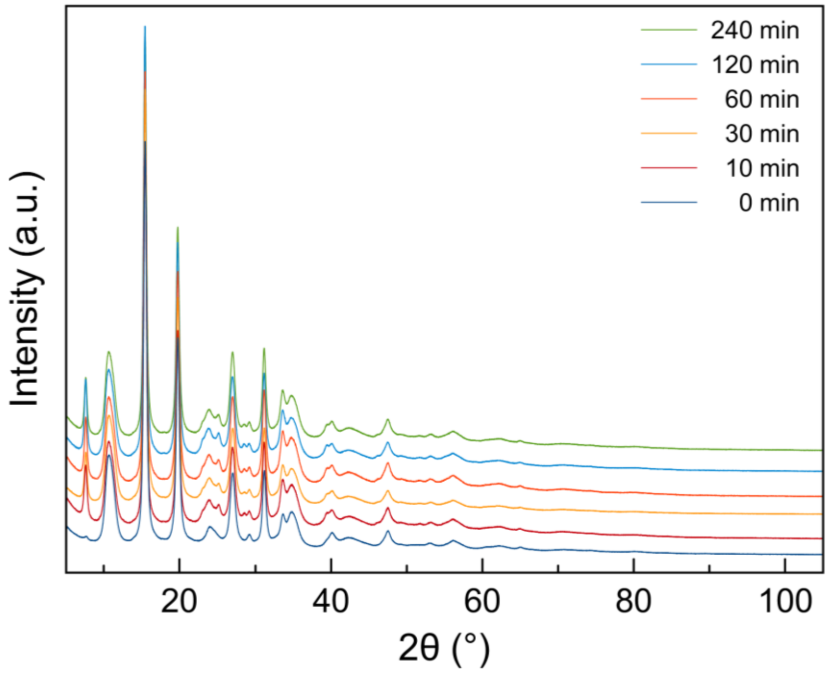

Figure S23. Powder X-ray diffraction patterns of the $HgCl_2$@ni-Zn/i-Zn samples recovered from the suspension of i-Zn in 500 ppm $HgCl_2$ aqueous solutions for different time lapses.

S10. Luminescence sensing of $HgCl_2$ in water

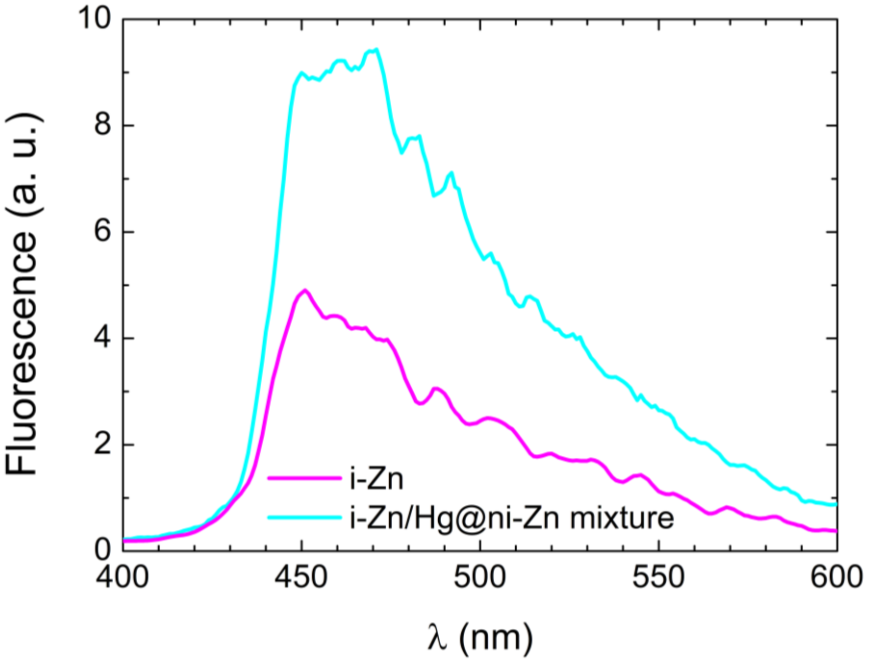

Figure S24. Fluorescence spectra of 1 μg/mL suspension of as-synthesized i-Zn (magenta line) and of the $HgCl_2$@ni-Zn/i-Zn system (cyan line) obtained upon suspending i-Zn in 500 ppm $HgCl_{2(aq)}$ for 1 h.

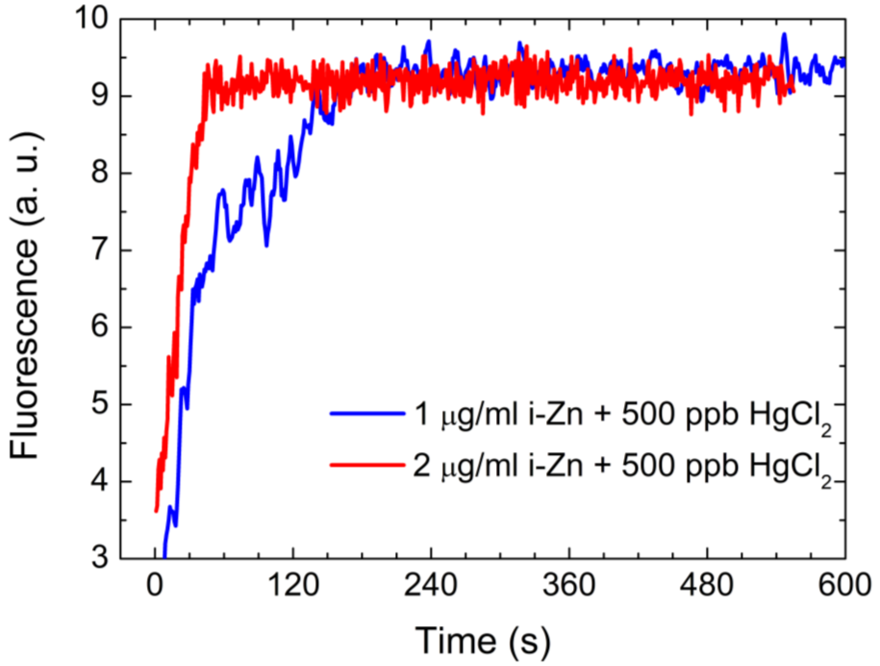

Figure S25. Fluorescence at 470 nm of suspensions of i-Zn in 500 ppb $HgCl_{2(aq)}$ versus suspension time. The i-Zn concentration is 1 μg/mL, equal to that used for building Figure 11 of the main text, in the blue curve, while it has been doubled in the red trace.

S11. References

(1) Lin, Q.; Meloni, D.; Pan, Y.; Xia, M.; Rodgers, J.; Shepard, S.; Li, M.; Galya, L.; Metcalf, B.; Yue, T.-Y.; Liu, P.; Zhou J. *Org. Lett.* **2009**, *11*, 1999–2002.

(2) Note that the 303-463 K value of $\Delta V/V_0$ reported in the text is normalized with respect to the 303 K unit cell parameters, while the values appearing in Figure S7b are normalized with respect to the tetragonal phase unit cell parameters detected at 483 K.

(3) Noyce, D. S.; Ryder jr., E.; Walker B. H. *J. Org. Chem.* **1995**, *20*, 1681-1686.

(4) Kistiakowsky, G. B. *Phys. Rev.* **1931**, *37*, 276-278.
